# Metabolic pathways underlying GATA6 regulating Trastuzumab resistance in Gastric Cancer cells based on untargeted metabolomics

**DOI:** 10.7150/ijms.50563

**Published:** 2020-10-23

**Authors:** Jinxia Chang, Qiang Wang, Anup Bhetuwal, Wenhu Liu

**Affiliations:** 1 School of Basic Medical Sciences, North Sichuan Medical College, Nanchong, Sichuan 637100, China.; 2 School of Pharmacy, North Sichuan Medical College, Nanchong, Sichuan 637100, China.; 3 Department of Laboratory Medicine, Affiliated Hospital of North Sichuan Medical College; Faculty of Laboratory Medicine, Center for Translational Medicine, North Sichuan Medical College, Nanchong, Sichuan 637000, China.; 4 Sichuan Key Laboratory of Medical Imaging and Department of Radiology, Affiliated Hospital of North Sichuan Medical College, Nanchong, Sichuan 637000, China.

**Keywords:** gastric cancer, trastuzumab resistance, GATA 6, untargeted metabolomics, TCA cycle

## Abstract

Trastuzumab has proven its effectiveness in gastric cancer with HER-2 gene-amplification, which has now developed resistance while the mechanism of which is not fully elucidated. Our previous studies demonstrated that the activity of GATA6 binding protein 6 (GATA6) enhanced prominently in trastuzumab resistant gastric cancer cell lines (NCI N87R and MKN45R). In the present study, we further confirmed the re-sensitization to trastuzumab and inhibition of mitochondrial functions of GATA6 knockout sublines (NCI N87R/ΔGATA6 and MKN45R/ΔGATA6). Moreover, we applied untargeted metabolomic profiling to investigate the potential roles of GATA6 in metabolism of NCI N87R and MKN45R. The UPLC system coupled with Q-Exactive Focus Orbitrap mass spectrometry, multivariate in combination with univariate analysis were performed for the screening of differential metabolites between resistant cells and GATA6 knockout sublines. A total of 68 and 59 endogenous metabolites were found to be altered significantly in NCI N87R/ΔGATA6 and MKN45R/ΔGATA6 cells compared with NCI N87R and MKN45R, respectively. Pathway analyses indicated disturbance of metabolic pathways after GATA6 knockout including tricarboxylic acid (TCA) cycle, glycolysis and energy-related amino acid pathways. An integrated proteomics-metabolomics revealed that sub-networks were closely related to TCA cycle, glycolysis, multiple amino acid and nucleotide metabolism. Western blot showed that TCA cycle and glycolysis-related molecules, including PKM, GLS, GLUL and LDHA, were downregulated in GATA6 knockout sublines. Taken together, these findings demonstrate that GATA6 is involved in metabolism reprogramming which might contribute to trastuzumab resistance in gastric cancer.

## Introduction

Gastric cancer, one of the common malignant tumors, is characterized by sudden occurrence, rapid progression, high morbidity and mortality 1. As part of the therapy, chemotherapy is widely applied in the treatment of gastric carcinoma, whilst drug resistance has become a primary cause of chemotherapy failure, which represents a significant challenge in the treatment of gastric cancer. Human epidermal growth factor receptor 2 (HER-2), a tyrosine kinase receptor assigned to the family of epidermal growth factor receptor (EGFR), is a crucial gastric cancer therapeutic target encoded by the *c-erbB2* proto-oncogene located on chromosome *17q21*^2^. HER-2 overexpression is associated with rapid tumor growth, metastasis as well as poor prognosis of gastric cancer while the activated HER-2 not only contributes to tumor survival signals but also facilitates reprogramming of cancer cells metabolism 3.

Trastuzumab, a monoclonal antibody targeting HER-2 receptor, has proven to be an effective agent for HER-2 positive advanced gastric cancer. However, the acquired resistance to trastuzumab impedes the curative effect and continuation of therapy. So far, the mechanisms underlying this resistance remain elusive. Hence, understanding the molecular mechanisms governing trastuzumab resistance has become of importance for seeking new therapeutic strategies.

Although the mechanisms of acquired resistance to chemotherapy in tumors are very complicated, missense mutations and metabolic reprogramming have become the most important and prioritized aspects of tumor chemotherapy failure. For instance, in pancreatic cancer disruption of glutamine metabolic pathways improves the efficacy of gemcitabine treatment 4, while HEATR1 deficiency promotes gemcitabine resistance by up-regulating Nrf2 signaling 5. Our previous studies have demonstrated that activation of Wnt/β-catenin/EMT pathways contributes to trastuzumab resistance in NCI N87 and MKN45 cells 6.

Transcription factor-mediated metabolic reprogramming has been implicated as a potential mechanism for drug resistance of cancer cells. GATA6, a zinc-finger transcription factor, functions as a tumor promoter or suppressor depending on the type of tumor. GATA6 overexpression has been observed in gastric cancer, breast cancer as well as in esophageal adenocarcinoma 7-9, while loss of GATA6 has been shown to be involved in malignant transformation of astrocytoma 10. Our recent proteomic study has shown that NCI N87R and MKN45R cells display a remarkable enhancement of GATA6 activity 11, whereas NCI N87R/ΔGATA6 and MKN45R/ΔGATA6 cells exhibit DNA impairment and glucose metabolism inhibition 12, suggesting that GATA6 knockout leads to an inhibition of energy metabolism. Our data therefore imply that blocking transcriptional activity of GATA6 could be an effective strategy to attenuate trastuzumab resistance in gastric cancer. Despite all of this, metabolic pathways and characteristics of GATA6 regulating trastuzumab resistance have yet to be elucidated in gastric cancer.

Metabolomics, a powerful tool to characterize complex biochemical systems, has been widely applied to life sciences fields, including illuminating the pathogenesis of cancers and mechanisms of drug resistance 13. In this study, we employed an untargeted metabolomics based on UPLC Q-Exactive Focus mass spectrometry to investigate metabolic pathways of GATA6 regulating trastuzumab resistance in gastric cancer cells. Pathway enrichment analysis suggested perturbation in pathways of TCA cycle, multiple amino acid metabolisms related to energy, glycolysis, and nucleotide metabolisms in NCI N87R/ΔGATA6 and MKN45R/ΔGATA6 cells. Additionally, we further analyzed the proteomic data in depth and constructed an integrated proteomics-metabolomics network of GATA6 regulating trastuzumab resistance to illuminate the regulatory relationships between metabolic pathways and the protein expression (proteomics data were found from our previous work 12). Our results showed that GATA6 knockout inhibited mitochondrial functions in NCI N87R and MKN45R cells, indicating that GATA6 could be a potential therapeutic target for dealing with trastuzumab resistance in gastric cancer.

## Materials and Methods

### Chemical and reagents

HPLC-grade acetonitrile, methanol, formic acid and ammonium acetate were supplied by CNW Inc. (Shanghai, China). 2-chloro-L-phenylalanine with a purity of > 98.5% was purchased from Heng-bai Biotech. Co. Ltd. (Shanghai, China). Deionized water was prepared from Millipore ultrapure water system (Merck Millopore, MA, USA).

### Cell lines and cell culture

Trastuzumab-resistant gastric cell lines (NCI N87R, MKN45R) and GATA6 knockout sublines (NCI N87R/ΔGATA6 and MKN45R/ΔGATA6) were constructed and preserved in our laboratory. All cells were cultured in DMEM medium (Gibco, NY, USA) supplemented with 10% fetal bovine serum (FBS) (Gibco, NY, USA), 100 units/mL penicillin and 100 μg/mL streptomycin and maintained at 37 °C in a humidified atmosphere containing 5% CO_2_-95% air. Cell lines were maintained in trastuzumab-containing medium (80 μg/mL) according to our previous procedures 6.

### Sample preparation for metabolome analysis

Intracellular metabolites extractions were performed according to methods that were utilized in our previous study 14. Briefly, cells were seeded in 6-well plates for 48-72 h in complete medium after which cells were harvested and counted at a density of 1×10^7^ cells/well. Next, the cells were washed twice with pre-cold PBS, and later, the metabolites were quenched and extracted with 1000 μL pre-cold mixed solvent (acetonitrile/methanol/water=2:2:1) containing 20 μL of 2-chloro-L-phenylalanine as the internal standard. After this, cell pellets were collected and transferred to a microcentrifuge tube, homogenized for 4 min and sonicated for 5 min in ice-water bath. This was followed by incubation at 20 °C for 1 h and centrifugation at 12 000 g for 15 min at 4 °C. The supernatants were filtered through 0.22 μm PTFE syringe filter. The filtrate was collected and transferred to autosampler vials for analysis by UPLC-Q Exactive Focus mass spectrometry. Quality control (QC) samples were prepared by pooling 30 μL of each sample in all groups. Solvent blank and QC samples were inserted in analytical batch after every five samples to assess the stability of the detecting system.

### UPLC Q-Exactive Focus mass spectrometry procedure

The metabolites were analyzed using an UPLC system (1290, Agilent) equipped with HSS T3 column (2.1 mm×100 mm, 1.8 μm, Waters, Milford, MA, USA). The temperature of the UPLC column oven was maintained at 35 °C while the autosampler was set at 10 °C. For the chromatographic separation, the mobile phase in positive ionization mode (ESI+) was composed of eluent A (0.1% formic acid in water, v/v) and eluent B (acetonitrile). Likewise, in negative ionization mode (ESI-) it was composed of eluent A (5 mM ammonium acetate in water) and eluent B (acetonitrile). The elution gradient was set as follows: 1% B from 0 to 1 minute; 1-99% B from 1 to 8 minute; 99% B from 8 to 10 minute; 99-1% B from 10 to 10.1 minute; 1% B from 10.1 to 12.0 minute. The elution flow rate was 0.5 ml/min. The injection volume was 5 μL.

High-resolution mass spectrometer Q-Exactive Focus Orbitrap MS mounted on the UPLC system was used to carry out the mass spectrometry equipped with dual electrospray ionization. The operating parameters were set as follows: The spray voltage was 4.0 kV in ESI+ and 3.6 kV in ESI-. The temperature of capillary was 320 °C, and aux gas heater was 350 °C, respectively. The aux gas and sheath gas flow rate were set at 15 arbitrary units and 30 psi, respectively. The full scan (MS1) range was 70-1000 m/z with a resolution of 70000. The automatic gain control (AGC) target for MS acquisitions was set to 1×10^6^ with a maximum ion injection time of 100 ms. Subsequent MS/MS scan (MS2) was processed with a resolution of 17500, and AGC target of 1×10^5^ with a maximum ion injection time of 60 ms. The dynamic exclusion time was 10 s. The mass spectra were analyzed at normalized collisional energy (NCE). Hight-purity nitrogen was used as the nebulizing gas and collision gas for higher energy collisional dissociation. Data acquisition was performed in the mode of information-dependent acquisition (IDA).

### Data transformation and metabolites identification

The acquired HPLC-MS raw files were converted to mzML files by using ProteoWizard. Subsequent data processing and analysis was accomplished using the bioinformatics program XCMS in R package (version 3.3.0) 15. The parameters settings were accomplished as it was in our previous study 14. OSI-SMMS software (version 1.0, Dalian Chem Data Solution Information Technology Co. Ltd.) was further used for peak annotation after XCMS data. The resulting generated a data that comprised of sample name, retention time (RT), mass-to-charge ratio (*m/z*), peak area and peak number. Metabolites were identified by matching MS/MS spectra fragmentation similarity with scores greater than or equal to 0.90 (the score range 0.00-1.00) based on self-built database containing more than 2000 endogenous metabolites.

### Multivariate statistical analysis and differential metabolic screening

Peak areas were normalized to the total peak area of each chromatogram. Processing of the missing values for each sample was performed with the 80 % rule while mean-centering and pareto-scaling were applied to reduce instrumental and chemical noise 16. Then multivariate statistical analysis was performed using MetaboAnalyst (version 4.0) for unsupervised principal component analysis (PCA) to observe natural cluster trend of the differential cell lines and QC samples 17. Subsequently, supervised orthogonal partial least square discriminant analysis (OPLS-DA) was implemented to maximize the differences of metabolic profiles between trastuzumab resistant groups and GATA6 knock out groups. The validation of all OPLS-DA models was assessed using 7-fold cross-validation and 200 permutation tests random iterations. The variable importance in the projection (VIP) plots from the OPLS-DA were applied to identify variables changed significantly in GATA6 knock out groups versus trastuzumab resistant groups. Variables with VIP values >1.0 were thought to be crucial which was further analyzed by independent *t*-tests. Volcano plots were accomplished by plotting the negative logarithm of *p*-value on the vertical axis (base 10) versus the logarithm of fold change (FC, base 2) on the horizontal axis. Fold change was calculated using average normalized peak areas in GATA6 knockout groups/resistant groups. SPSS 19.0 software was implemented for statistical analysis of the normalized values to determine metabolites with significant changes. Variables with VIP >1.0, FC ≥ 1.2 or ≤ 0.83 and *p* value < 0.05 were regarded as differentially altered metabolites. Data were displayed as mean ±SEM and presented by GraphPad Prism 8.0.1 (GraphPad Prism, Inc. San Diego, USA).

### Integrated analysis of metabolomics and proteomics

MetaboAnalyst (version 4.0) was applied to explore biological functions of differential metabolites which set the cut-off of pathway impact value from the topology analysis to 0.1 as according to previous methods 17. The Kyoto Encyclopedia of Genes and Genomes database (KEGG, version 89.1) was explored to investigate the disturbed metabolic pathways. OmicsNet database (version 1.0) was utilized to acquire the network analysis and molecular interactions with the standard setting 18. Cytoscape software (version 3.7.2) was applied to visualize the network models from differential metabolites and regulatory target genes.

### Western blotting assay

Western blot analysis was performed according to protocols described recently 6. Briefly, cells from different groups were lysed using RIPA lysis buffer (50 mM Tris, 150 mM NaCl, 1% Triton X-100, 1% sodium deoxycholate, 0.1% sodium dodecyl sulfate, Beyotime, China) containing 1% protease inhibitor. Lysates were centrifuged for 15 min at 4 °C (12000 g), after which the supernatant was collected for further use. Proteins concentration were quantified with the Broadford assay kit (CWBIO, China). Equal amounts (20 µg) of proteins were denatured by heating and separated by SDS-PAGE, followed by transfer to nitrocellulose membranes which were later incubated with designated primary antibodies against PKM (Abcam, #38237, UK), GLS2 (Abcam, #113509, UK), GLUL (Abcam, #49873, UK), LDHA (Abcam, #125683, UK) and *β*-actin (Cell Signaling, #5176, USA) (dilution 1:1000) respectively at 4 °C overnight after blocking with 5% skimmed milk. Thereafter, membranes were incubated with suitable secondary antibodies (ZSGB-Bio, China) (dilution 1:3000) at room temperature. Finally, chemiluminescence signals were visualized using an enhanced chemiluminescence reagent (CWBIO, China). The grey values of these signals were measured using Image J and histograms were plotted using GraphPad Prism 8.0.1.

### Cell viability assays

Cell viability was assessed using CCK-8 kit according to previous protocols 6. Cells were seeded in a 96-well plate at 4×10^3^ cells/well and incubated for 24 h. The cells were then treated with a range of indicated concentrate of trastuzumab (0, 20, 40, 80, 160, 320 and 640 μg/mL) for 72 h. Absorbance was detected at 450 nm with a microplate reader (Bio-Rad, Hercules, CA, USA). All data were represented based on three independent experiments.

### Mitochondria isolation and purification

Mitochondria isolation were executed using a Cell Mitochondria Isolation Kit (Beyotime, #C3601, China) according to the manufacturer's instructions. Briefly, 5×10^7^ cells were prepared and washed twice with pre-cold PBS and centrifuged at 800 g for 5 min at 4 °C. Thereafter, cells were homogenized 50 times using a homogenizer with the mitochondria isolation buffer supplemented with 1 mM protease inhibitor. The cell homogenates were then centrifuged at 1000 g for 10 min at 4 °C. The supernatant was transferred to 1.5-mL tubes and further centrifuged at 3500 g for 10 min at 4 °C. The mitochondria pellet was resuspended with the mitochondria storage buffer for further analysis.

### Detection of mitochondrial membrane potential

Mitochondrial membrane potential was determined using Mitochondrial Membrane Potential Assay Kit with JC-1 (Beyotime, #C2006, China). Purified mitochondria were mixed with JC-1 buffer (1×). Fluorescence intensity was detected with Multi-Mode Reader (BioTek) at an excitation of 485 nm and emission wavelength of 535 nm for JC-1 monomers and an excitation of 535 nm and emission wavelength of 595 nm for J-aggregates. The mitochondrial membrane potential was presented as the ratio of J-aggregates to monomers.

### Detection of the cellular adenosine triphosphate in mitochondria

ATP levels in mitochondria were detected using an ATP Assay Kit (Beyotime, #S0027, China) according to the manufactures' instructions. 100 μL ATP working reagent was added to a 96-well plate and put at room temperature for 5 min and then added with 20 μL of purified mitochondria per-well. The ATP levels were tested using a Multi-mode reader (BioTek) and calculated according to the standard ATP curve.

## Results

### GATA6 knockout re-sensitized resistant cells to trastuzumab

**Figure 1:**
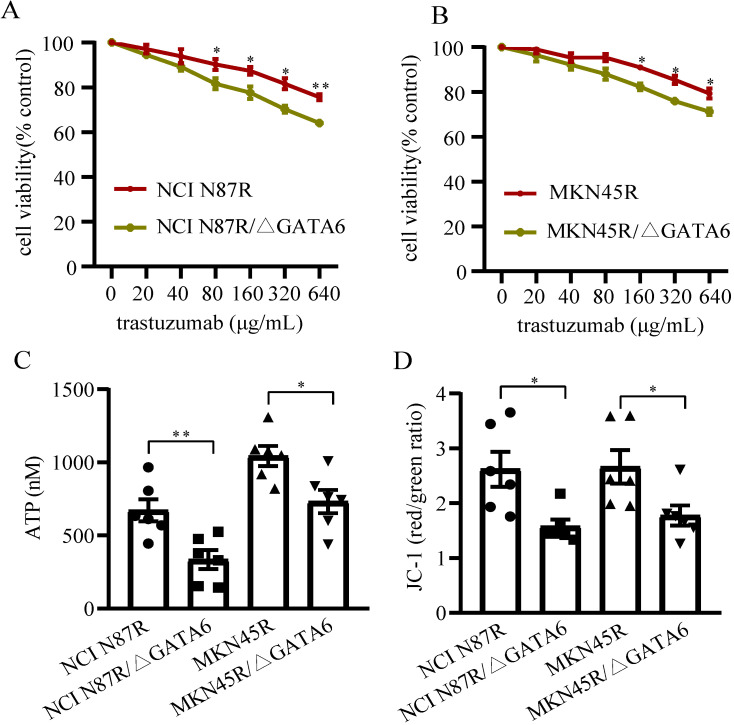
** Detection of cell viability and mitochondrial function.** (**A and B**) Cells were incubated with different concentrations of trastuzumab for 72 h, and then cell viability was measured by CCK-8 kit. (**C**) ATP levels were tested using ATP assay Kit. (**D**) MMPs were detected using Mitochondrial Membrane Potential Assay Kit with JC-1. Six independent biological replicates are shown as mean±SEM. ^*^p<0.05, ^**^p<0.01 *vs*. controls.

To investigate if GATA6 knockout decreased trastuzumab resistance in gastric cancer cells, NCI N87R, NCI N87R/ΔGATA6, MKN45R and MKN45R/ΔGATA6 cells were cultured under indicated concentrations of trastuzumab for 72 h. The results showed trastuzumab inhibited viability of NCI N87R, NCI N87R/ΔGATA6, MKN45R and MKN45R/ΔGATA6 cells in a dose-dependent manner, while the more distinct inhibitory effects were observed in NCI N87R/ΔGATA6 and MKN45R/ΔGATA6. It reached significance from a statistical standpoint at 80 μg/mL with cell viability of 90.25% and 81.62% in NCI N87R and NCI N87R/ΔGATA6, and 160 μg/mL with cell viability of 88.98% and 82.37% in MKN45R and MKN45R/ΔGATA6 cells, respectively (Fig. 1A, B). These results demonstrated that GATA6 knockout re-sensitized resistant cells to trastuzumab.

### GATA6 knockout inhibited mitochondrial function of trastuzumab resistant gastric cancer cells

We also detected the MMP and ATP levels between trastuzumab resistant groups and GATA6 knockout groups, respectively. The results displayed a relatively lower levels of ATP and MMP in GATA6 knockout groups compared to control groups (Fig. 1C, D), suggesting that knockout of GATA6 inhibited mitochondrial function and affected energy metabolism of trastuzumab resistant cells.

### Quality control analysis of mass spectrometric data

**Figure 2:**
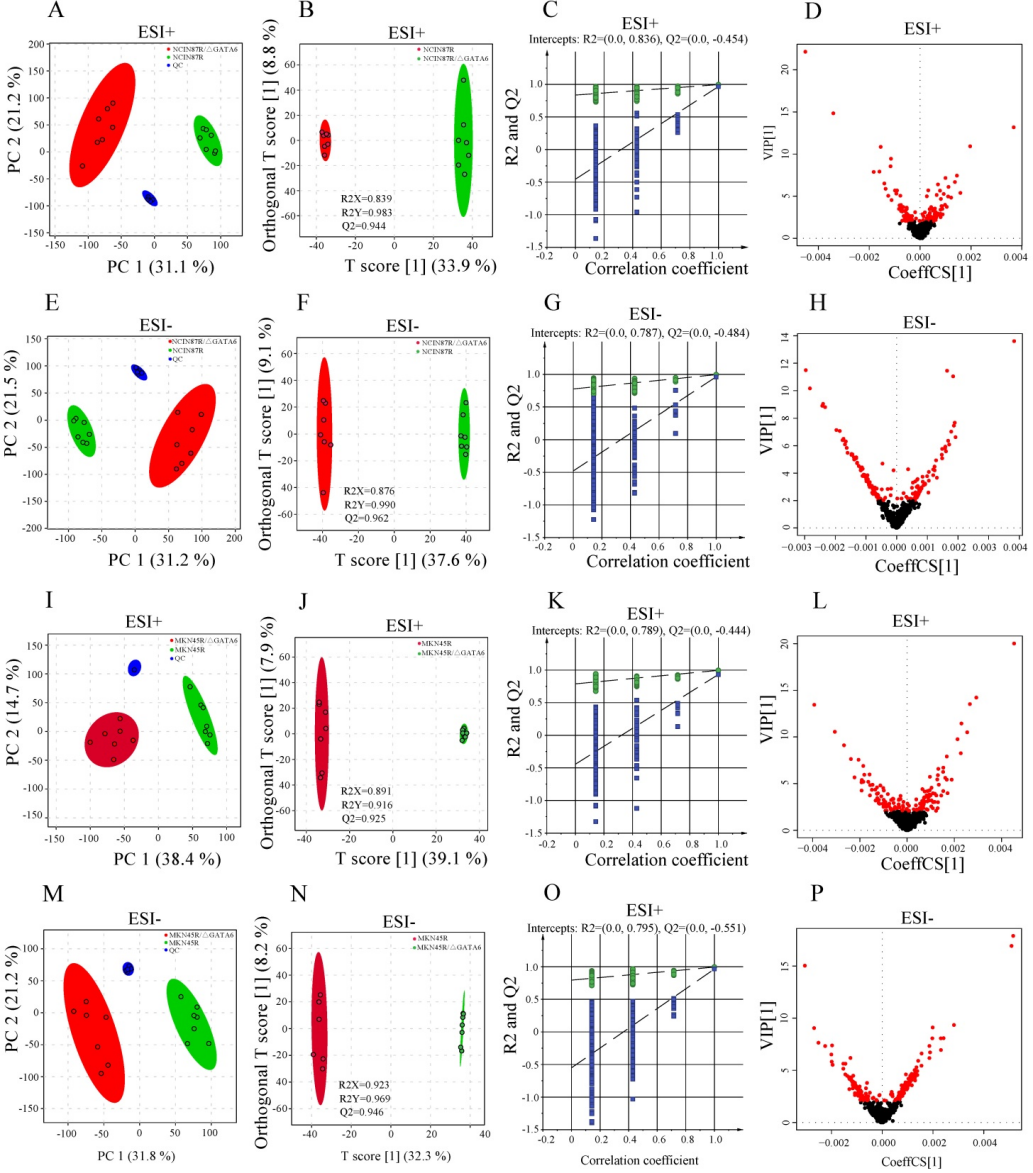
Differential metabolomics profiling between NCI N87R and NCI N87R/ΔGATA6, MKN45R and MKN45R/ΔGATA6 cells. (**A, E, I, M**) PCA score plots of NCI N87R/ΔGATA6 and NCI N87R cells, MKN45R/ΔGATA6 and MKN45R cells in ESI+ and ESI-, respectively. (**B, F, J, N**) OPLS-DA score plots of NCI N87R/ΔGATA6 and NCI N87R cells, MKN45R/ΔGATA6 and MKN45R cells in ESI+ and ESI-, respectively. (**C, G, K, O**) Permutation test plots of NCI N87R/ΔGATA6 and NCI N87R cells, MKN45R/ΔGATA6 and MKN45R cells in ESI+ and ESI-, respectively. (**D, H, L, P**), VIP plots of NCI N87R/ΔGATA6 and NCI N87R cells, MKN45R/ΔGATA6 and MKN45R cells in ESI+ and ESI- respectively, red nodes represent corresponding variables with VIP > 1.0 in each group.

To evaluate the quality of the mass spectrometric data, pooled QC samples and standard compound (2-chloro-L-phenylalanine) were applied to monitor the stability of the LC/MS systems and control the reproducibility of the sample treatment procedures. The results indicated that QC samples were clustered tightly in PCA scatter plot (Fig. 2A, E, I, M) with relative standard deviations (RSD%) of peak areas being 3.24% in positive ionization mode (ESI+) and 4.79% in negative ionization mode (ESI-), respectively (Table S1). PCA-X one-dimensional score plots displayed good reproducibility in trastuzumab resistant groups and GATA6 knockout groups with both positive and negative ion ionization modes within ±2 standard deviation (Std), respectively (Fig. S1). Moreover, the high correlation (*r*>0.90) of peak areas from QC samples demonstrated a good reproducibility (Fig. S2). Clearly, these results indicated a satisfactory stability of the data acquisition system.

### Metabolomics profiling of trastuzumab resistant gastric cancer cells and trastuzumab resistant gastric cancer cells with GATA6 knockout

To disclose the metabolic behaviors between trastuzumab resistant gastric cancer cells with GATA6 knockout and trastuzumab resistant cells, an untargeted metabolomics-based strategy was applied to profile the metabolites in ESI+ and ESI-, respectively. Total of 3990 and 3894 features were detected from NCI N87R/ΔGATA6, NCI N87R and MKN45R/ΔGATA6, MKN45R cells in ESI+, whereas 4335 and 4216 features were obtained in ESI-, respectively. These features matched successfully with 73 (ESI+) and 68 (ESI-) metabolites in NCI N87R/ΔGATA6 and NCI N87R cells, along with 66 (ESI+) and 59 (ESI-) metabolites in MNK45R/ΔGATA6 and MKN45R cells and associated with MS2 scores more than 0.90 (score range: 0.00-1.00). PCA was performed at first to discern the presence of variable differences in mass spectral profiles between trastuzumab resistant groups and GATA6 knockout groups in both ion modes. The apparent differences indicated intrinsic variations in trastuzumab resistant cells with GATA6 knockout versus trastuzumab resistant cells (Fig. 2A, E, I, M). Next, a supervised OPLS-DA was employed to distinguish the differences further as evident from the score plots which showed notable separation between different groups without overlap (Fig. 2B, F, J, N). The results of the OPLS-DA modes were explained by variance R2X (0.839 and 0.891 for ESI+, 0.876 and 0.923 for ESI- in NCI N87R/ΔGATA6 and MKN45R/ΔGATA6, respectively), R2Y (0.983 and 0.916 for ESI+, 0.990 and 0.969 for ESI- in NCI N87R/ΔGATA6 and MKN45R/ΔGATA6, respectively), and predicted variance Q2 (0.944 and 0.925 for ESI+, 0.962 and 0.946 for ESI- in NCI N87R/ΔGATA6 and MKN45R/ΔGATA6, respectively). This indicated the classification models had a good explanatory and predictive ability for all models. To prevent model overfitting, the validity of OPLS-DA models was further analyzed using 200 iterations permutation tests with intercepts values R2=0.836, Q2 =-0.454 (for ESI+), and R2=0.787, Q2=-0.484 (for ESI-) in NCI N87R/ΔGATA6 cells (Fig. 2C and G), respectively. Similarly validity in MKN45R/ΔGATA6 cells were evaluated with intercepts values R2=0.789, Q2=-0.444 (for ESI+), and R2=0.795, Q2=-0.551 (for ESI-), respectively (Fig. 2K and O). These results revealed that OPLS-DA model was reliable and not over-fitted. Next, a heatmap was applied to visualize the whole metabolome comparison of both the groups, which indicated a significant change of the metabolome (Fig. S3).

### Identification of differential metabolites

**Figure 3:**
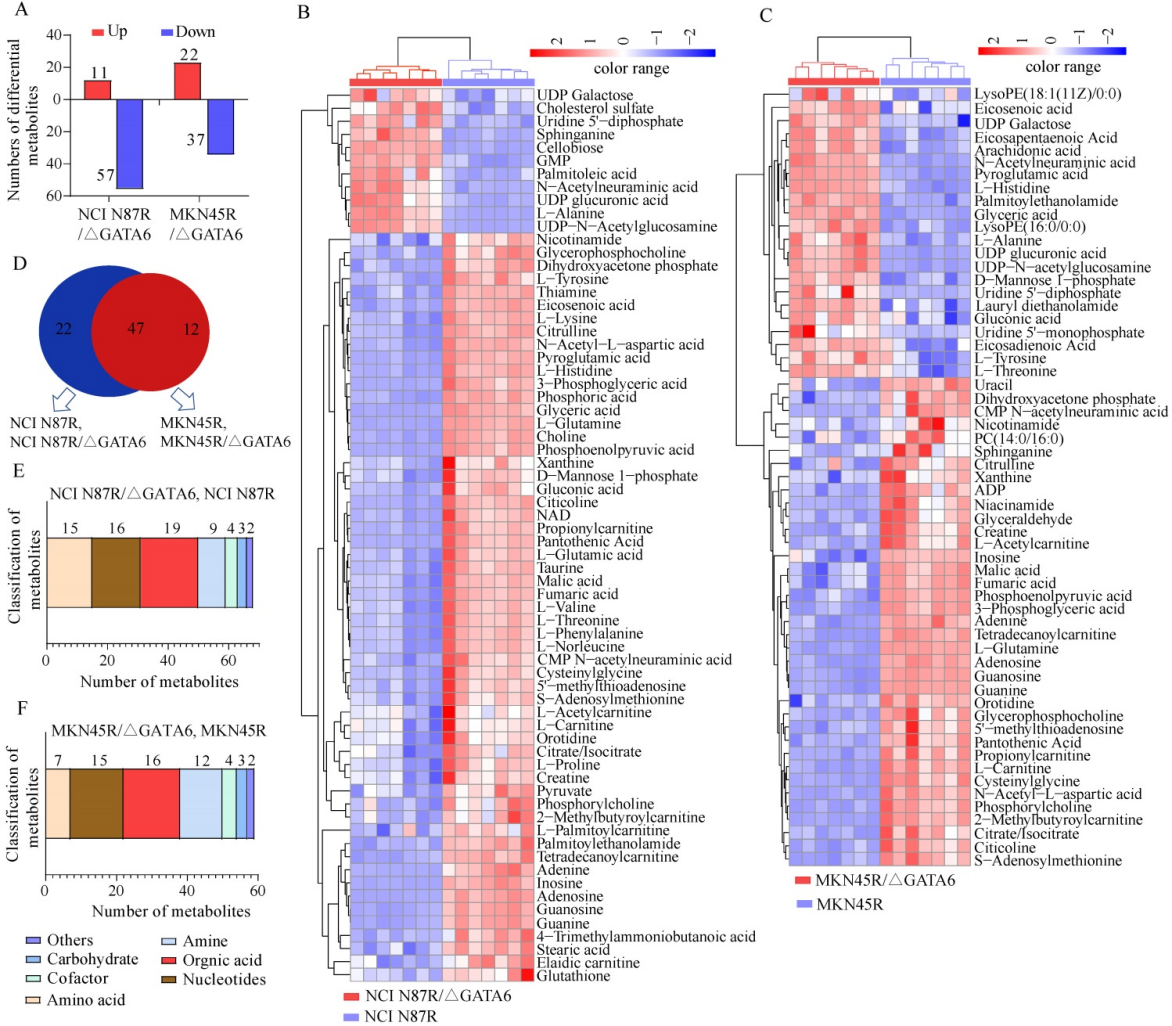
Analysis of differential metabolites between GATA6 knock out groups *vs*. trastuzumab resistant groups. (**A**) Increased and decreased metabolites in NCI N87R/ΔGATA6 and MKN45R/ΔGATA6 cells. (**B, C**) Heat map visualization of differential metabolites. Each row represents a metabolite and each column represents a sample. The color scale with red and blue intensity denotes increased and decreased metabolites, respectively. (**D**) Venn diagram shows the shared and non-shared metabolites among NCI N87R/ΔGATA6, NCI N87R cells, MKN45R/ΔGATA6 and MKN45R cells. (**E, F**) Classification of differential metabolites by their properties.

**Figure 4:**
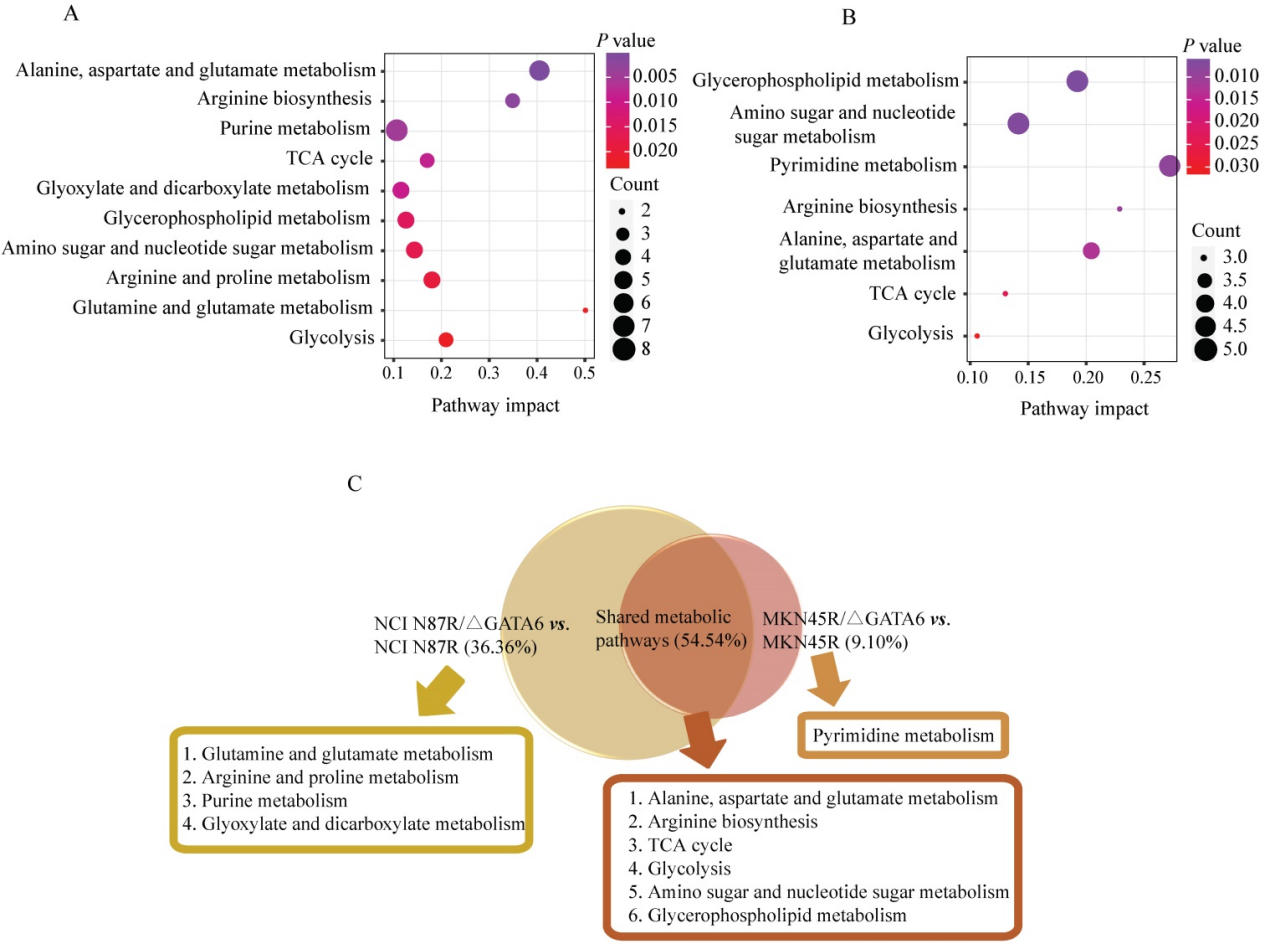
Pathway enrichment analysis corresponding to the differential metabolites via MetaboAnalyst in (**A**) NCI N87/ΔGATA6 and NCI N87R, (**B**) MKN45/ΔGATA6 and MKN45R cells. x-axis represents the pathway impact value of topological analysis. Size of the nodes shows the number of matched metabolites, color of the nodes indicates *p* value of the enrichment analysis.

Based on the OPLS-DA models, VIP-plots were explored to analyze critical variables that contributed to distinguishing the metabolome between GATA6 knockout groups and resistant groups in both ion modes (Fig. 2D, H, L, P). Those variables with VIP value>1 were considered as potential candidates of significance. Subsequently, volcano plots were used to screen differential metabolite abundances against the corresponding *p* value obtained from unpaired-sample *t*-test (Fig. 4A-D). Differential metabolites were confirmed by matching retention time and MS2 fragmentation patterns (MS2 scores ≥ 0.90) from self-built database. Compared with NCI N87R and MKN45R cells, the levels of 11 and 22 metabolites displayed a more than 1.2 fold increase in NCI N87R/ΔGATA6 and MKN45R/ΔGATA6 cells (VIP>1 and *p*<0.05), respectively. Similarly, 57 and 37 metabolites showed a less than 0.83 fold decrease in NCI N87R/ΔGATA6 and MKN45R/ΔGATA6 cells (VIP>1, *p*<0.05), respectively (Fig. 3A, Tables 1 & 2). A hierarchical cluster visualization of differential metabolites indicated that majority of metabolites sharply decreased in GATA6 knock out groups versus resistant groups (Fig. 3B, C). From the Venn diagram, it was determined that 47 metabolites overlapped in NCI N87R/ΔGATA6 and MKN45R/ΔGATA6 cells (Table S2), however, 22 identified metabolites exclusively were of NCI N87R and NCI N87R/ΔGATA6 cells (Fig. 3D, Table S3), whereas 12 metabolites were exclusive of MKN45R and MKN45R/ΔGATA6 cells (Fig. 3D, Table S4). These metabolites were further classified according to their properties, including amino acids, amines, organic acids, nucleotides, cofactors, carbohydrates and others, of which organic acid, amino acid and nucleotides accounted for more than half of all identified metabolites (Fig. 3E, F).

### Metabolic pathways analysis

**Figure 5:**
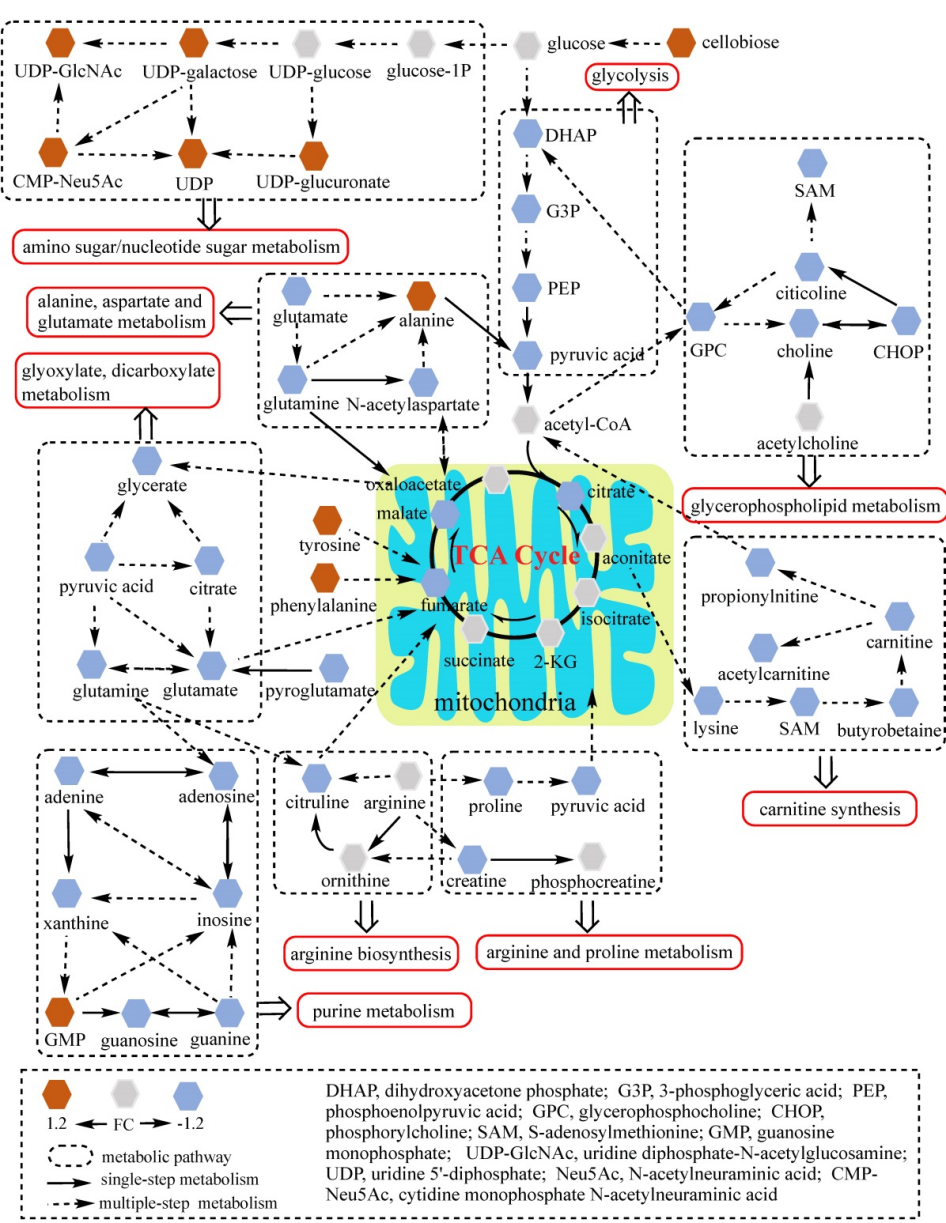
Network analysis of significantly changed metabolic pathways. Ten pathways disturbed in NCI N87R/ΔGATA6 cells were analyzed. Each hexagon represents a metabolite, brown and blue ones represent 1.2 fold increase or decrease in NCI N87R/ΔGATA6 cells respectively. Dashed box indicates different metabolic pathways. Solid arrows show single-step metabolism, and dotted arrows show multiple-step metabolism.

**Figure 6:**
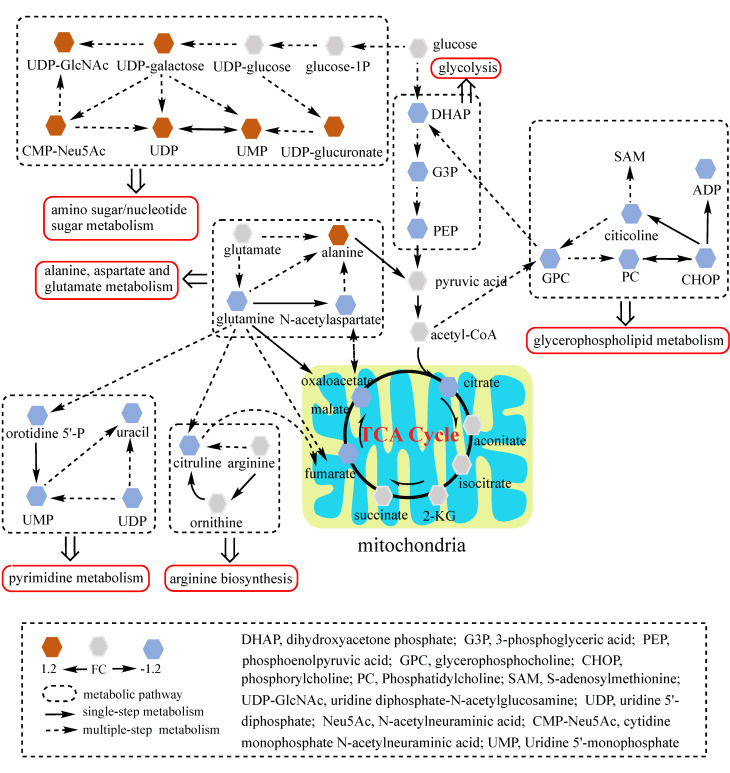
Network analysis of significantly changed metabolic pathways. Seven pathways disturbed in MKN45R/ΔGATA6 cells were analyzed. Each hexagon represents a metabolite, brown and blue ones represent 1.2 fold increase or decrease in MKN45R/ΔGATA6 cells respectively. Dashed box indicates different metabolic pathways. Solid arrows show single-step metabolism, and dotted arrows show multiple-step metabolism.

To better understand the underlying mechanisms of trastuzumab resistance attenuated by GATA6 knockout in gastric cancer cells, all differential metabolites in NCI N87R/ΔGATA6 and MKN45R/ΔGATA6 cells were imported into MetaboAnalyst (version 4.0) for metabolic analysis, respectively. Accordingly, the pathway library of *Homo sapiens* and Fisher's exact test were applied for pathway enrichment analysis, and relative-betweeness centrality was performed for pathway topology analysis based on reported protocols 17. The pathway impact values were calculated using cumulative percentage according to matched metabolites, and *p* values were acquired by enrichment analysis based on false FDR. The influenced metabolic pathways were set as pathway impact values more than 0.10 and *p* value less than 0.05. According to the *p* and pathway impact values, ten metabolic pathways were observed in NCI N87R/ΔGATA6 cells (Fig. 4A, Table 3). Among them, three pathways were involved in carbohydrate metabolism: (I) citrate cycle, (II) amino sugar and nucleotide sugar metabolism and (III) glycolysis. Four pathways were related to amino acid metabolism: (I) glutamine and glutamate, (II) arginine biosynthesis, (III) arginine and proline and (IV) alanine, aspartate and glutamate metabolism. Moreover, one pathway was subjected to lipid metabolism which involved glycerophospholipid metabolism. We also found that purine metabolism was particularly altered but possessed a low impact value (*p*=0.004, pathway impact value=0.101). Notably, among these pathways, alanine, aspartate and glutamate metabolism displayed the lowest *p* value (*p*=9.71×10^-5^), whereas glutamine and glutamate metabolism showed highest pathway impact value (pathway impact value=0.50). Correspondingly, seven pathways showed prominent changes in MKN45R/ΔGATA6 cells compared to MKN45R cells (Fig. 4B, Table 4). Out of which, three metabolic pathways were relevant to carbohydrate metabolism pathways: (I) glycolysis, (II) amino sugar and nucleotide sugar metabolism, and (III) TCA cycle. Two pathways were part of amino acid metabolism: (I) arginine biosynthesis, and (II) alanine, aspartate and glutamate metabolism. Similarly, glycerophospholipid metabolism was also observed in MKN45R/ΔGATA6 cells. In contrast, rather than purine metabolism, pyrimidine metabolism showed a significant change solely in MKN45R/ΔGATA6 cells. Apparently, as it can be interpreted from the Venn diagram, six disturbed metabolic pathways were shared by both NCI N87R/ΔGATA6 and MKN45R/ΔGATA6 cells, accounting for 54.54% of all pathways altered in these two groups. These pathways include (I) alanine, aspartate and glutamate metabolism, (II) amino sugar and nucleotide sugar metabolism, (III) arginine biosynthesis, (IV) glycerophospholipid metabolism, (V) glycolysis, and (VI) TCA cycle (Fig. 4C). Conversely, some pathways exhibited distinct characteristics as a result of pyrimidine metabolism which was noticeably changed in MKN45R/ΔGATA6 cells. However, some pathways such as glyoxylate and dicarboxylate metabolism along with arginine and proline metabolism occurred exclusively in NCI N87R/ΔGATA6 cells (Fig. 4C). Next, KEGG database (version 89.1) was employed to portray integrated metabolic pathway map to reveal the most relevant metabolic pathways and their potential functions in GATA6 knock out groups based on pathway enrichment and topology analysis. The results were presented manually by drawing united metabolic pathway networks (Figs. 5 & 6). Noticeably, all metabolic pathways were strongly associated with those related to energy metabolism such as TCA cycle, amino acid metabolism, glycolysis and nucleotide metabolism, indicating that GATA6 modulate the energy metabolism by regulating various metabolic pathways. Collectively, these disturbed metabolic pathways have afforded a deep insight of the mechanisms involved in trastuzumab resistance of gastric cancer cells.

### Integrated analysis of metabolomics and proteomics

**Figure 7:**
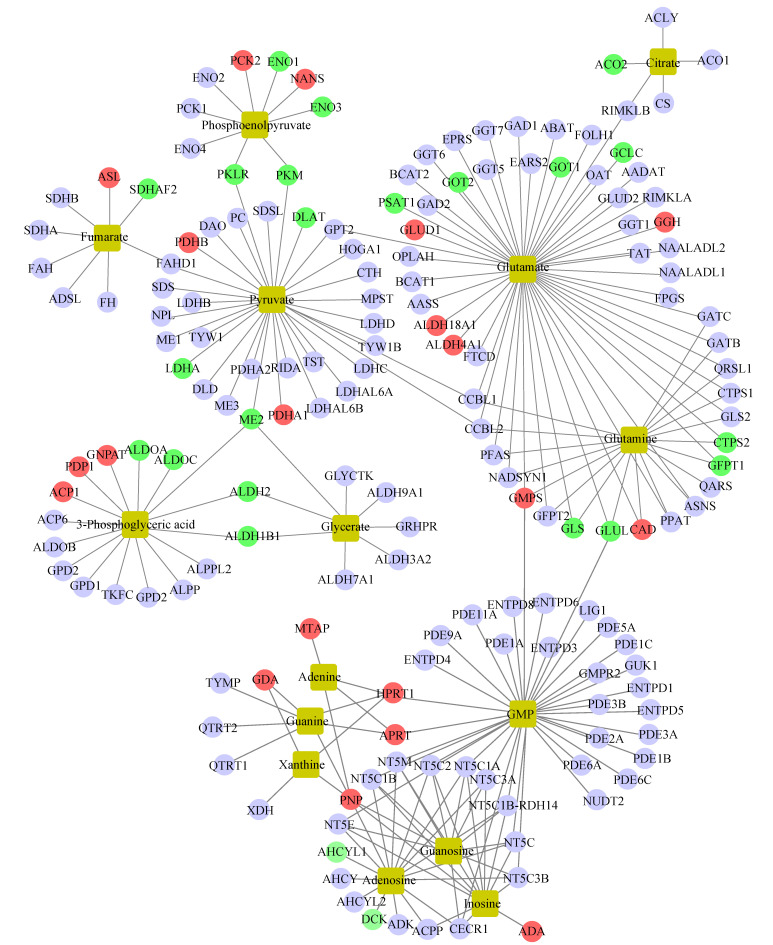
Network analysis of differential metabolites and activated transcriptional factors based on the OmicsNet. Red and green nodes represent up- and down- regulated transcriptional factors, respectively. Light blue nodes indicate transcriptional factors with no significant changes in NCI N87R/ΔGATA6 cells compared to NCI N87R. Yellow nodes represent increased and decreased metabolites respectively. Lines represent interaction between transcription factors and metabolites.

**Figure 8:**
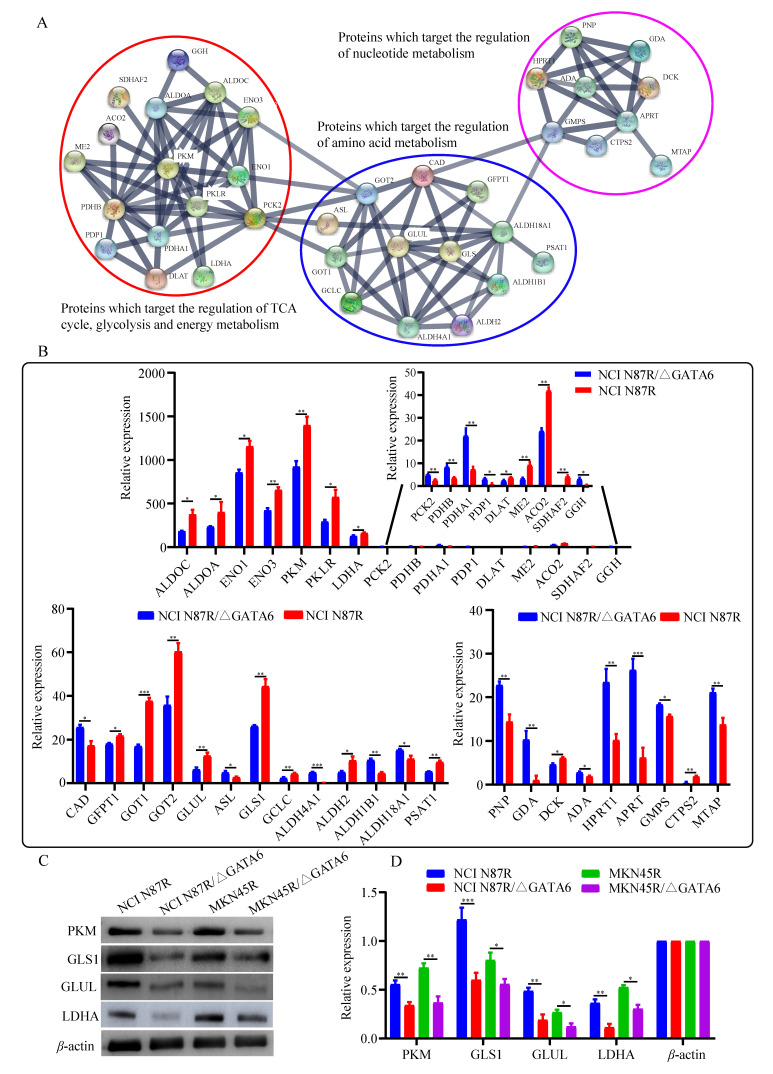
Bioinformatics and quantification analysis of activated transcription factors. (**A**) Network analysis of activated transcription factors done with Cytoscape software. (**B**) Quantification of activated transcription factors by mass spectrometry. (**C**) PKM, GLS1, GLUL and LDHA detected using western blot with *β*-actin as loading control. (**D**) Quantification of western blot signals in two groups of cell lines. Three independent biological replicates are shown as mean±SEM. ^*^p<0.05, ^**^p<0.01, ^**^p<0.001 *vs*. controls.

To further unveil the potential metabolism-related mechanisms responsible for GATA6-mediated trastuzumab resistance, we constructed an integrated metabolite-protein regulatory network from differential metabolites and target genes based on the OmicsNet (version 1.0) 18, from which the results were annotated using a visual network diagram (Fig. 7). We observed that fifteen crucial differential metabolites were closely related to TCA cycle, energy metabolism and nucleotide metabolism, including phosphoenolpyruvate, citrate/isocitrate, fumarate, pyruvate, glutamate, glutamine, 3-phosphoglyceric acid (3-PG), glycerate, adenine, guanine, xanthine, GMP, guanosine, adenosine and inosine. Phosphoenolpyruvate, which is involved in glycolysis and gluconeogenesis, exhibited a dramatic decrease in both NCI N87R/ΔGATA6 and MKN45R/ΔGATA6 cells. Furthermore, integrated data showed that phosphoenolpyruvate was regulated by nine molecules including PCK2, ENO1, NANS, ENO3, PKM, PKLR, ENO4, PCK1 and ENO2. Among them, PCK2 and NANS were increased, whereas conversely, ENO1, ENO3, PKM and PKLR were decreased in NCI N87R/ΔGATA6 cells. Similarly, thirty target proteins regulate pyruvate, another key intermediate metabolite in carbohydrate, protein and fat metabolism that regulates sugar metabolism out of which PKLR, PKM, DLAT, LDHA and ME2 were downregulated while PDHB, PDHA1 were upregulated in NCI N87R/ΔGATA6 cells. We also noticed that RKLR and PKM were co-regulatory molecules in pyruvate-phosphoenolpyruvate sub-network. Similarly, fumarate, a precursor to L-malate in TCA cycle, which is converted by the enzyme fumarase to malate, was regulated by SDHB, SDHA, FAH, ADSL, FH, SDHAF2, ASL and FAHD1. From these molecules only ASL displayed an obvious up-regulation, whereas, SDHAF2 showed a distinct down-regulation in NCI N87R/ΔGATA6 cells. Additionally, we observed that five different molecules regulated citrate with only ACO2 showing significant down-regulation whereas no significant changes occurred in the expression of CS, ACO1, ACLY and RIMKLB in NCI N87R/ΔGATA6 cells, indicating that ACO2 plays a major role in regulating citrate. Moreover, we noticed that 3-PG, which is a significant metabolic intermediate in both glycolysis and TCA cycle, reduced sharply in GATA6 knock out cells. It was regulated by some typical molecules including ACP1, PDP1, GNPAT, ALDOA, ALDOC, ALDH2, ALDH1B1 and ME2, which were all altered significantly. Meanwhile, we found eight different molecules modulated glycerate, out of which ALDH2, ALDH1B1 and ME2 showed prominent down-regulation in NCI N87R/ΔGATA6 cells. According to the sub-network, we also observed that ME2 is a co-regulatory molecule of pyruvate, 3-PG and glycerate. Besides, when compared with NCI N87R cells, our data revealed some factors that regulate glutamate and glutamine. GLUD1, GGH, ALDH18A1, ALDH4A1, GMPS and CAD displayed a prominent up regulation, in contrast, GCLC, GOT1, GOT2, PSAT1, CTPS2, GFPT1, GLS and GLUL showed dramatic down regulation in NCI N87R/ΔGATA6 cells. Several molecules, which regulate adenine, guanine, xanthine, GMP, guanosine, adenosine and inosine, were also changed dramatically in which AHCYL1 and DCK were decreased, while MTAP, GDA, HPRT1, APRT, PNP and ADA were increased instead (Fig. 7). Next, we employed the STRING database (version 11.0) to construct interactive networks of activated transcription factors in NCI N87R/ΔGATA6 cells, whose interactions have high confidence with scores more than 0.70 19. We noticed that PKM and PKLR represented primary hubs in regulating TCA cycle, glycolysis and energy metabolism, GLUL and GLS displayed another center in regulating amino acid metabolism, and APRT represented a main hub in regulating nucleotide metabolism (Fig. 8A). Quantification of these transcription factors using proteomics are shown in Fig. 8B. Thereafter, we confirmed the expression alteration of PKM, GLS1, GLUL and LDHA in NCI N87R/ΔGATA6 and MKN45R/ΔGATA6 cells using western blotting, and consistent with our mass spectrometry data, a remarkable down regulation of PKM, GLUL, GLS1 and LDHA were observed in GATA6 knock out groups as compared to their resistant groups (Fig. 8C, D).

## Discussion

TCA cycle, considered as the central hub for supervision of energy metabolism for cancer cells, contributes to constant supply of energy for synthesis of proteins, lipid and nucleic acids. Abnormal TCA cycle is involved in several biological processes of cancer cells including aberrant metabolism 20. Our results showed that TCA cycle was suppressed and led to abnormal energy metabolism as evidenced by a distinct decrease in fumarate, malate and citrate/isocitrate in GATA6 knock out cells (Figs. S5, S6). It is worth noting that relatively lower levels of ATP and MMP were observed in GATA6 knockout groups compared to trastuzumab resistant groups (Fig. 1C, D). Furthermore, proteomics data displayed that expression of mitochondrial aconitase (ACO2), which is an important regulatory enzyme of the TCA cycle, was significantly downregulated in NCI N87R/ΔGATA6 cells. Similarly, succinate dehydrogenase assembly factor 2 (SDHAF2), which is a component of both the TCA cycle and the mitochondrial electron transport chain, was also decreased in NCI N87R/ΔGATA6 cells when compared with NCI N87R cells. It is well-known that NAD-dependent malic enzyme (ME2) plays a key role in the malate/aspartate shuttle across the mitochondrial membrane involving regulation of redox balance, cellular energy and biosynthesis of molecules. Various studies have demonstrated that ME2 overexpression is associated with cell migration and invasion, whereas low expression of ME2 leads to a decrease in synthesis efficiency of DNA, thereby ultimately causing cancer cells apoptosis 21, 22. The present study showed that GATA6 knockout resulted in the down-regulation of ME2 in trastuzumab resistant gastric cancer cells, which in turn reduced ATP production and ultimately inhibited the proliferation of cells. Taken together, these results suggest that GATA6 contributes to trastuzumab resistance, which is dependent on TCA cycle and mitochondrial function in gastric cancer.

Cancer cells have higher rate of glycolysis than normal cells in order to generate more ATP for metabolic activities, which is a hallmark of cancer termed as “Warburg effect” and has been widely accepted as a common feature of metabolic reprogramming 23. Increasing body of evidence has revealed that inhibition of glycolysis in cancer cells is an effective approach to overcome multidrug resistance related to mitochondrial respiratory defect and hypoxia 24. Multiple molecules involved in glycolysis have been reported to be associated to HER-2 signaling pathway in cancer cells, such as lactate dehydrogenase A (LDHA), a key enzyme in the glycolytic pathway, the level of which is facilitated by overexpression of HER-2 which further enhances the utilization of glucose and decreases the consumption of oxygen in breast cancer cells 25. Accordingly, inhibition of LDHA activity compromised the tumorigenesis and proliferation of HER-2 initiated cancer cells 26. Moreover, the high level of glutamine synthetase (GLUL) was positively correlated with the expression of HER-2 and proliferation of cancer cell 27. Of note, reduced glucose uptake and hexokinase activity were also observed in HER-2 positive breast cancer cells following treatment with trastuzumab 28. However, present studies are mainly focused on the interplay between HER-2 and glycolysis in the initiation and progression of breast cancers. Hence, the role of HER-2 signaling in the aberrant glucose metabolism of gastric cancer remains to be elucidated.

In this study, we found that some key metabolites related to glycolysis showed a dramatic decrease in GATA6 knockout groups, including dihydroxyacetone (DHAP), G3P, phosphoenolpyruvate (PEP), and pyruvic acid (Figs. S5, S6), suggesting that GATA6 is one of the key factors in the regulation of glycolysis in trastuzumab resistant gastric cancer cells. Pyruvate kinase M (PKM) is known to be an important rate-limiting enzyme of the glycolytic pathway, which catalyzes the conversion of PEP and adenosine diphosphate (ADP) into pyruvate and ATP. A previous study has suggested that low level of GATA6 triggers glycolysis by activating PKM in hepatocellular carcinoma 29. However, unlike the above study, our data showed that GATA6 knockout caused down-regulation of PKM and inhibition of glycolysis. This discrimination might be due to the different cell type (hepatocellular carcinoma vs. gastric cancer). However, other molecular mechanisms should also be taken into account. Knockdown of LDHA in tumor cells induces increased mitochondrial respiration, decreased proliferation and suppressed tumorigenicity 30. Previous study showed that LDHA has a relatively higher expression in paclitaxel-resistant than paclitaxel- sensitive breast cancer cells, and down-regulation of LDHA can re-sensitizes resistant cells to paclitaxel again 31. In consistency with this study, we also found that GATA6 knockout led to decrease of LDHA, and subsequently glycolysis was further inhibited in NCI N87R/ΔGATA6 and MKN45R/ΔGATA6 cells. Collectively, it might suggest that GATA6 confers glycolysis by regulating glycolytic related kinase activity and expression. Moreover, we found that amino sugar and nucleotide sugar metabolism was promoted due to increased uridine 5'-diphosphate-galactose (UDP-galactose), uridine diphosphate-N-acetylglucosamine (UDP-GlcNAc), N-acetylneuraminic acid (UDP-Neu5Ac), uridine diphosphate (UDP) and UDP-glucuronate levels in the cells with GATA6 knock out. We propose that this might be an alternative mechanism of GATA6 knock out cells, primarily activating the amino sugar and nucleotide sugar metabolism to compensate for the inhibited glycolytic pathway for acquiring necessary energy and nutrients.

Mounting evidence has shed light on the important roles of amino acids in metabolism of cancer cells 32. It is known that glutamate is a nitrogen donor for synthesizing some nitrogenous compounds in cancer cells. Glutamine is a major amino acid that drives the TCA cycle to sustain mitochondrial ATP production in cancer cells 33. The utilization of glutamine decreased drastically when the cells were under hypoxia or with mitochondrial dysfunction 34. Proline is a source of carbon exchange between TCA cycle and urea cycle. Arginine serves as the precursor to proline or as an additional source of glutamate. Our results demonstrated lower levels of glutamine, glutamate, N-acetyl-aspartate, citrulline and proline while higher levels of alanine, tyrosine, and phenylalanine in trastuzumab resistant cells with GATA6 knockout compared with trastuzumab resistant gastric cancer cells, suggesting that GATA6 plays an important role in amino acid metabolisms of trastuzumab resistant gastric cancer cells. Of note, these altered metabolites belong to glutamate and glutamine metabolism, alanine, aspartate and glutamate metabolism, arginine and proline metabolism, and arginine biosynthesis (Tables 3 & 4). Furthermore, we analyzed two critical enzymes regulating glutamine and glutamate. Aspartate aminotransferase (GOT1/2), important regulators of levels of glutamate which contributes to glucose synthesis and gluconeogenesis, have been found to be upregulated in various tumor cells 35. Moreover, in colorectal cancer cell, inhibition of GOT1 activity increases the sensitivity to 5-fluorouracil 36. Our study showed that GOT1 and GOT2 were decreased in trastuzumab resistant gastric cancer cells with GATA6 knockout, suggesting that GATA6 maintains basal expression of GOT1 and GOT2. Glutaminase (GLS), which catalyzes the transformation of glutamine to glutamate, is regarded as another critical enzyme in growth and proliferation of cancer cells. A prior study has shown that silencing of GLS leads to re-sensitization of taxol-resistant breast cancer, suggesting its key role in taxol-resistance 37. Consistently, we found that GATA6 knockout resulted in decreased expression of GLS and thereupon disturbed glutamate and glutamine metabolism in trastuzumab resistant gastric cancer cells. Furthermore, several amino acid metabolic pathways were altered in NCI N87R/ΔGATA6 cells including arginine and proline metabolism and arginine biosynthesis. Collectively, we speculated that GATA6 regulates multiple amino acid metabolism pathways, which might contribute to trastuzumab resistance, while the underlying mechanisms remain to be further investigated.

Nucleotide metabolism is a key pathway that generates purine and pyrimidine molecules for DNA replication and synthesis. It is well known that enhanced nucleotide metabolism contributes to growth of tumors 38. Pyrimidines, along with purine metabolism, are two avenues of nucleotide metabolism that produce metabolites including adenine, adenosine, inosine and guanosine monophosphate (GMP), etc. The present study showed that GATA6 knockout led to suppressed purine metabolism since relevant metabolites including adenine, adenosine, xanthine, guanosine, inosine, glutamine, guanine and orotidine-5P were sharply decreased in NCI N87R/ΔGATA6 cells. In MKN45R/ΔGATA6 cells, pyrimidine metabolism was found to be inhibited. It was widely accepted that glutamine is one of the important precursors for *de novo* nucleotides synthesis as it provides nitrogen required for purine and pyrimidine synthesis 39. GLUL, which catalyzes the ATP-dependent conversion of glutamate and ammonia to glutamine, was dramatically downregulated upon GATA6 knockout in our study. A study reported that high expression of GLUL affects cellular response to irradiation in radiation-resistant cells and facilitates growth of cancer cells 40. Thus, it might suggest that nucleotide metabolism mediated by GATA6 also contributes to trastuzumab resistance in gastric cancer cells.

In conclusion, our study demonstrated that GATA6 is involved in TCA cycle, glycometabolism, amino acid and nucleotide metabolism, thereby leading to reprogramming in the metabolism and promoting trastuzumab resistance in gastric cancer cells. Recovering the abnormal metabolism of GATA6 in gastric cancer cells could be a potential therapeutic strategy for dealing with trastuzumab resistance.

## Supplementary Material

Supplementary figures and tables.

## Figures and Tables

**Table 1:** Significantly changed metabolites in NCI N87R/ΔGATA6 compared with NCI N87R cells

No.	Name	Adduct	Observed mass	Exact mass	Mass error (ppm)	RT(s)^a^	VIP value^b^	FC^c^	*p* value	ESI^d^
1	Proline	[M+H] +	116.0705	115.0633	-6.9	35.42	4.58	0.59	5.74×10^-5^	+
2	Cellobiose	[M+Na] +	365.1047	342.1162	-1.5	33.79	1.03	8.39	2.76×10^-14^	+
3	Choline	[M]+	104.1069	104.1075	-5.8	32.47	2.97	0.40	5.87×10^-11^	+
4	5'-Methylthioadenosine	[M+H] +	298.0963	297.0896	-4.4	35.84	5.21	0.62	3.67×10^-5^	+
5	Adenine	[M+H] +	136.0617	135.0545	-5.9	44.45	1.36	0.09	2.69×10^-7^	+
6	Creatine	[M+H] +	132.0767	131.0695	-6.1	35.70	2.09	0.71	1.36×10^-3^	+
7	Thiamine	[M]+	265.1113	265.1123	-3.8	36.48	11.73	0.50	2.51×10^-8^	+
8	Guanosine	[M+H] +	284.0984	283.0917	-4.6	135.82	1.05	0.21	1.61×10^-10^	+
9	Valine	[M-H] -	116.0719	117.0789	8.5	35.95	1.75	0.56	6.28×10^-6^	-
10	Lysine	[M+H] +	147.1127	146.1055	-5.4	28.71	1.35	0.58	3.44×10^-8^	+
11	Citrulline	[M-H] -	174.0886	175.0957	5.1	31.66	2.75	0.44	4.21×10^-9^	-
12	Pyroglutamic acid	[M+H] +	130.0498	129.0425	-5.4	91.95	1.29	0.24	6.72×10^-11^	+
13	Histidine	[M+H] +	156.0767	155.0695	-5.1	30.32	1.01	0.39	6.43×10^-10^	+
14	GMP	[M+H] +	364.0644	363.0579	-4.1	56.79	1.53	2.84	3.11×10^-9^	+
15	Glutamic acid	[M+H] +	148.0603	147.0532	-6.1	32.34	1.57	0.51	3.97×10^-6^	+
16	Taurine	[M+H] +	126.0219	125.0147	-6.4	31.12	1.39	0.58	1.16×10^-6^	+
17	Citicoline	[M+H] +	489.1135	488.1073	-3.6	35.33	1.09	0.20	1.49×10^-8^	+
18	Pantothenic acid	[M-H] -	218.1036	219.1107	4.1	44.69	2.98	0.11	1.29×10^-7^	-
19	Niacinamide	[M+H] +	123.0552	122.0480	-6.5	40.89	1.44	0.74	6.37×10^-3^	+
20	Acetylcarnitine	[M+H] +	204.1229	203.1157	-3.9	56.96	2.19	0.76	3.95×10^-2^	+
21	Phosphorylcholine	[M]+	184.0732	184.0738	-3.2	56.18	1.89	0.57	9.40×10^-4^	+
22	Glycerophosphocholine	[M+H] +	258.1098	257.1028	-3.9	31.77	1.17	0.49	2.22×10^-6^	+
23	Palmitoylethanolamide	[M+H] +	300.2892	299.2824	-4.0	480.69	4.38	0.33	4.47×10^-8^	+
24	Carnitine	[M+H] +	162.1123	161.1052	-5.6	34.33	1.12	0.76	1.41×10^-2^	+
25	S-Adenosylmethionine	[M]+	399.1437	399.1451	-3.5	36.27	1.19	0.53	1.56×10^-4^	+
26	Tyrosine	[M-H] -	180.0669	181.0739	5.5	56.96	2.17	0.42	7.40×10^-7^	-
27	Propionylcarnitine	[M+H] +	218.1384	217.1314	-4.6	133.78	1.24	0.32	4.16×10^-7^	+
28	Cysteinylglycine	[M+H] +	179.0484	178.0412	-4.5	44.91	1.51	0.32	2.55×10^-5^	+
29	NAD	[M+H] +	664.1149	663.1091	-3.3	56.79	2.40	0.46	3.45×10^-6^	+
30	2-Methylbutyroylcarnitine	[M+H] +	246.1697	245.1627	-4.1	196.15	1.63	0.64	1.82×10^-3^	+
31	Guanine	[M+H] +	152.0566	151.0494	-5.3	135.84	2.84	0.22	9.57×10^-11^	+
32	Palmitoylcarnitine	[M+H] +	400.3412	399.3349	-4.2	613.81	5.53	0.65	2.32×10^-2^	+
33	N-Acetyl-L-aspartic acid	[M-H] -	174.0409	175.0481	4.6	25.90	1.30	0.42	2.25×10^-9^	-
34	Tetradecanoylcarnitine	[M+H] +	372.3101	371.3036	-4.0	541.29	2.47	0.31	4.31×10^-9^	+
35	4-Trimethylammoniobutanoic acid	[M+H] +	146.1175	145.1103	-5.5	177.67	1.27	0.78	2.43×10^-5^	+
36	Glutamine	[M+H] +	147.0763	146.0691	-5.5	31.57	4.17	0.10	1.60×10^-14^	+
37	Adenosine	[M+H] +	268.1036	267.0968	-4.5	135.80	1.02	0.02	2.62×10^-11^	+
38	Sphinganine	[M+H] +	302.3047	301.2980	-4.3	534.68	1.97	2.94	4.67×10^-7^	+
39	Elaidic carnitine	[M+H] +	426.3569	425.3505	-3.8	645.00	1.49	0.79	4.41×10^-4^	+
40	Xanthine	[M-H] -	151.0263	152.0334	5.9	58.40	1.64	0.34	1.79×10^-3^	-
41	Stearic acid	[M-H] -	283.2645	284.2715	3.5	547.56	2.97	0.64	4.31×10^-6^	-
42	Phosphoenolpyruvic acid	[M-H] -	166.9752	167.9823	5.4	24.85	1.30	0.18	1.63×10^-10^	-
43	Citric/Isocitric acid	[M-H] -	191.0199	192.0270	4.7	25.72	2.46	0.64	1.75×10^-3^	-
44	Malic acid	[M-H] -	133.0144	134.0215	6.7	25.33	2.22	0.59	1.44×10^-6^	-
45	Glutathione	[M-H] -	306.0745	307.0838	-4.2	26.62	1.25	0.69	2.02×10^-2^	-
46	Palmitoleic acid	[M-H] -	253.2176	254.2245	4.3	452.15	2.16	1.62	5.51×10^-5^	-
47	Glyceric acid	[M-H] -	105.0194	106.0266	7.5	27.38	1.10	0.09	3.80×10^-11^	-
48	Mannose 1-phosphate	[M-H] -	259.0226	260.0297	3.4	25.72	1.26	0.52	1.94×10^-4^	-
49	Dihydroxyacetone phosphate	[M-H] -	168.9909	169.9980	5.3	26.00	1.25	0.49	3.76×10^-7^	-
50	Phosphoric acid	[M-H] -	96.9697	97.9769	8.2	26.94	1.88	0.52	5.17×10^-10^	-
51	Inosine	[M-H] -	267.0738	268.0808	3.7	133.96	1.57	0.08	2.83×10^-11^	-
52	Phenylalanine	[M-H] -	164.0719	165.0789	6.1	138.26	1.51	0.51	9.47×10^-7^	-
53	Eicosenoic acid	[M-H] -	309.2802	310.2871	3.5	553.02	1.33	0.39	1.34×10^-10^	-
54	3-Phosphoglyceric acid	[M-H] -	184.9858	185.9929	4.9	25.46	1.44	0.08	2.54×10^-8^	-
55	UDP Galactose	[M-H] -	565.0484	566.0550	2.5	26.29	1.63	1.59	1.99×10^-3^	-
56	Threonine	[M-H] -	118.0512	119.0582	8.4	31.39	1.02	0.50	1.09×10^-6^	-
57	Alanine	[M-H] -	88.0404	89.0476	9.0	44.24	1.28	8.18	1.22×10^-7^	-
58	Norleucine	[M-H] -	130.0875	131.0946	6.9	56.43	1.59	0.47	5.26×10^-7^	-
59	Uridine 5'-diphosphate	[M-H] -	402.9955	404.0021	3.4	33.92	1.54	1.88	3.37×10^-5^	-
60	Pyruvic acid	[M-H] -	87.0087	88.0160	7.9	30.59	1.92	0.63	2.29×10^-2^	-
61	Orotidine	[M-H] -	287.0524	288.0594	3.5	30.02	1.75	0.67	2.52×10^-3^	-
62	CMP N-acetylneuraminic acid	[M-H] -	613.1405	614.1473	1.9	26.01	2.03	0.53	2.07×10^-5^	-
63	UDP glucuronic acid	[M-H] -	579.0277	580.0343	2.4	23.59	2.64	2.89	1.55×10^-5^	-
64	N-Acetylneuraminic acid	[M-H] -	308.0990	309.1059	3.6	28.03	1.98	1.75	5.51×10^-6^	-
65	UDP-N-acetylglucosamine	[M-H] -	606.0751	607.0815	2.6	27.07	3.06	6.12	1.28×10^-8^	-
66	Cholesterol sulfate	[M-H] -	465.3048	466.3116	2.6	446.27	1.57	1.27	7.72×10^-5^	-
67	Fumaric acid	[M-H] -	115.0038	116.0109	7.8	25.15	2.26	0.54	1.84×10^-7^	-
68	Gluconic acid	[M-H] -	195.0512	196.0583	4.6	27.64	2.49	0.34	4.36×10^-5^	-

a. RT, retention time; b. VIP, variable importance in projection; c. FC, fold change (NCI N87R/ΔGATA6 *vs*. NCI N87R); d. ESI, electrospray ionization.

**Table 2:** Significantly changed metabolites in MKN45R/ΔGATA6 compared with MKN45R cells

No.	Name	Adduct	Observed mass	Exact mass	Mass error (ppm)	RT (s)^a^	VIP value^b^	FC^c^	*p* value	ESI^d^
1	5'-methylthioadenosine	[M+H] +	298.0963	297.0895	-4.0	35.84	2.25	0.70	3.73×10^-5^	+
2	Adenine	[M+H] +	136.0617	135.0545	-5.9	44.44	15.42	0.45	1.73×10^-8^	+
3	Creatine	[M+H] +	132.0767	131.0695	-6.1	35.70	2.78	0.73	2.81×10^-5^	+
4	Guanosine	[M+H] +	284.0984	283.0917	-4.6	135.82	7.96	0.16	2.38×10^-12^	+
5	Niacinamide	[M+H] +	123.0552	122.0480	-6.6	59.26	3.15	0.62	7.93×10^-5^	+
6	Pyroglutamic acid	[M+H] +	130.0498	129.0425	-5.4	31.94	3.84	1.76	5.57×10^-12^	+
7	Histidine	[M+H] +	156.0767	155.0695	-5.2	30.31	1.79	1.52	1.91×10^-9^	+
8	Citicoline	[M+H] +	489.1135	488.1073	-3.7	35.33	1.36	0.62	2.02×10^-6^	+
9	Nicotinamide	[M+H] +	123.0552	122.0480	-6.6	40.88	2.39	0.76	0.02	+
10	Acetylcarnitine	[M+H] +	204.1229	203.1157	-3.9	56.95	2.78	0.61	2.79×10^-5^	+
11	Phosphorylcholine	[M]+	184.0732	184.0738	-3.3	56.18	1.86	0.50	1.09×10^-7^	+
12	Glycerophosphocholine	[M+H] +	258.1098	257.1028	-3.9	31.76	3.14	0.76	1.74E^-5^	+
13	Palmitoylethanolamide	[M+H] +	300.2892	299.2824	-4.0	480.69	1.67	1.71	3.47×10^-9^	+
14	Carnitine	[M+H] +	162.1123	161.1052	-5.6	34.32	1.75	0.58	2.22×10^-8^	+
15	S-Adenosylmethionine	[M]+	399.1437	399.1451	-3.5	36.27	1.70	0.58	4.07×10^-7^	+
16	Tyrosine	[M+H] +	182.0811	181.0739	-4.4	70.53	1.08	1.28	0.002	+
17	Propionylcarnitine	[M+H] +	218.1384	217.1314	-4.6	133.77	2.23	0.62	1.62×10^-6^	+
18	Cysteinylglycine	[M+H] +	179.0484	178.0412	-4.5	44.90	5.83	0.59	1.99×10^-7^	+
19	2-Methylbutyroylcarnitine	[M+H] +	246.1697	245.1627	-4.1	196.15	1.46	0.46	1.20×10^-8^	+
20	Guanine	[M+H] +	152.0566	151.0494	-5.3	135.83	2.94	0.18	2.53×10^-13^	+
21	Lauryl diethanolamide	[M+H] +	288.2529	287.2460	-3.8	379.18	2.05	1.43	3.37×10^-5^	+
22	Tetradecanoylcarnitine	[M+H] +	372.31	371.3036	-4.3	541.29	1.07	0.07	5.93×10^-11^	+
23	Glutamine	[M+H] +	147.0763	146.0691	-5.5	31.566	7.06	0.08	8.91×10^-15^	+
24	PC (14:0/16:0)	[M+H] +	706.5376	705.5308	-1.7	380.78	1.72	0.71	5.03×10^-3^	+
25	Adenosine	[M+H] +	268.1036	267.0968	-4.5	135.79	2.32	0.09	9.63×10^-12^	+
26	Sphinganine	[M+H] +	302.3047	301.2980	-4.3	534.68	2.06	0.72	1.02×10^-2^	+
27	LysoPE (16:0/0:0)	[M+H] +	454.2919	453.2855	-3.5	416.32	1.35	1.53	2.87×10^-7^	+
28	LysoPE (18:1(11Z)/0:0)	[M+H] +	480.3076	479.3012	-3.3	428.31	1.16	1.28	3.1×10^-2^	+
29	Citric/Isocitric acid	[M-H] -	191.0199	192.0270	4.7	27.65	2.58	0.68	2.04×10^-5^	-
30	Xanthine	[M-H] -	151.0263	152.0334	5.9	58.40	4.10	0.52	8.04×10^-3^	-
31	Phosphoenolpyruvic acid	[M-H] -	166.9752	167.9823	5.4	24.84	1.50	0.23	6.66×10^-8^	-
32	Malic acid	[M-H] -	133.0144	134.0215	6.7	25.33	3.12	0.78	4.22×10^-6^	-
33	Eicosadienoic Acid	[M-H] -	307.2646	308.2715	3.6	517.33	2.52	1.59	0.02	-
34	Glyceric acid	[M-H] -	105.0194	106.0266	7.5	27.37	4.40	2.74	1.14×10^-11^	-
35	Mannose 1-phosphate	[M-H] -	259.0226	260.0297	3.5	25.72	9.64	1.45	2.10×10^-3^	-
36	Dihydroxyacetone phosphate	[M-H] -	168.9909	169.9980	5.3	26.00	2.31	0.57	6.37×10^-5^	-
37	Inosine	[M-H] -	267.0738	268.0808	3.7	133.96	7.21	0.45	1.03×10^-3^	-
38	N-Acetyl-L-aspartic acid	[M-H] -	174.0409	175.0481	4.6	25.90	2.26	0.69	9.48×10^-9^	-
39	Pantothenic Acid	[M-H] -	218.1036	219.1107	4.1	44.68	2.10	0.63	7.28×10^-6^	-
40	Uridine 5'-monophosphate	[M-H] -	323.0288	324.0358	3.1	29.44	9.26	1.90	0.01	-
41	Eicosenoic acid	[M-H] -	309.2802	310.2872	3.2	553.01	1.65	1.43	0.02	-
42	3-Phosphoglyceric acid	[M-H] -	184.9858	185.9929	4.9	25.46	2.12	0.16	2.06×10^-9^	-
43	Glyceraldehyde	[M-H] -	89.0245	90.0316	10	28.46	1.49	0.75	1.12×10^-4^	-
44	UDP-galactose	[M-H] -	565.0484	566.0550	2.5	26.29	1.01	1.55	6.48×10^-5^	-
45	Threonine	[M-H] -	118.0512	119.0582	8.4	31.39	4.74	1.28	8.37×10^-5^	-
46	Eicosapentaenoic Acid	[M-H] -	301.2176	302.2245	3.6	432.09	4.45	1.65	1.51×10^-6^	-
47	ADP	[M-H] -	426.0227	427.0294	3.0	41.64	2.07	0.57	2.37×10^-5^	-
48	Alanine	[M-H] -	88.0404	89.0476	9.0	44.24	1.88	1.42	1.49×10^-5^	-
49	Uridine 5'-diphosphate	[M-H] -	402.9955	404.0022	3.2	33.92	1.74	2.13	5.0×10^-4^	-
50	Arachidonic acid	[M-H] -	303.2333	304.2402	3.6	462.07	4.68	1.67	3.67×10^-7^	-
51	Orotidine	[M-H] -	287.0524	288.0594	3.5	30.01	5.26	0.57	1.03×10^-5^	-
52	CMP N-acetylneuraminic acid	[M-H] -	613.1405	614.1473	1.9	26.00	1.97	0.70	1.57×10^-5^	-
53	UDP glucuronic acid	[M-H] -	579.0277	580.0343	2.4	23.58	1.23	2.34	4.93×10^-9^	-
54	N-Acetylneuraminic acid	[M-H] -	308.0990	309.1059	3.6	28.02	1.88	1.65	1.40×10^-9^	-
55	UDP-N-acetylglucosamine	[M-H] -	606.0751	607.0815	2.6	27.06	3.17	1.86	8.43×10^-10^	-
56	Uracil	[M-H] -	111.0201	112.0273	7.1	31.07	6.44	0.59	0.003	-
57	Fumaric acid	[M-H] -	115.0038	116.0109	7.8	25.14	2.15	1.24	1.74×10^-6^	-
58	Gluconic acid	[M-H] -	195.0512	196.0583	4.6	27.64	3.73	1.29	0.001	-
59	Citrulline	[M-H] -	174.0886	175.0956	5.7	31.65	1.44	0.71	0.002	-

a. RT, retention time; b. VIP, variable importance in projection; c. FC, fold change (MKN45R/ΔGATA6 *vs*. MKN45R); d. ESI, electrospray ionization.

**Table 3:** Ten prominent metabolic pathways enriched via MetaboAnalyst based on differential metabolites identified in NCI N87R/ΔGATA6 *vs*. NCI N87R cells

No.	Pathway name	Hits/Total	*p* values	Pathway impact values
1	Alanine, aspartate and glutamate metabolism	7/28	9.71×10^-5^	0.399
2	Arginine biosynthesis	4/14	2.06×10^-3^	0.345
3	Purine metabolism	8/65	4.65×10^-3^	0.101
4	TCA cycle	4/20	8.22×10^-3^	0.166
5	Glyoxylate and dicarboxylate metabolism	5/32	9.29×10^-3^	0.111
6	Glycerophospholipid metabolism	5/36	1.53×10^-2^	0.121
7	Amino sugar and nucleotide sugar metabolism	5/37	1.71×10^-2^	0.139
8	Arginine and proline metabolism	5/38	1.91×10^-2^	0.176
9	Glutamine and glutamate metabolism	2/6	2.33×10^-2^	0.50
10	Glycolysis	4/26	2.21×10^-2^	0.206

**Table 4:** Seven prominent metabolic pathways enriched via MetaboAnalyst based on differential metabolites identified in MKN45R/ΔGATA6 *vs*. MKN45R cells

No.	Pathway name	Hits/Total	*p* values	Pathway impact values
1	Alanine, aspartate and glutamate metabolism	4/28	1.27×10^-2^	0.203
2	Arginine biosynthesis	3/14	9.98×10^-3^	0.228
3	Pyrimidine metabolism	5/65	8.47×10^-3^	0.270
4	TCA cycle	3/20	2.43×10^-2^	0.129
5	Glycerophospholipid metabolism	5/36	5.98×10^-3^	0.190
6	Amino sugar and nucleotide sugar metabolism	5/37	6.75×10^-3^	0.139
7	Glycolysis	3/26	3.16×10^-2^	0.106

## Abbreviations

3-PG: 3-phosphoglyceric acid
ACLY: ATP-citrate synthase
ACO1: cytoplasmic aconitate hydratase
ACO2: aconitate hydratase
ACP1: low molecular weight phosphotyrosine protein phosphatase
ADA: adenosine deaminase
ADSL: adenylosuccinate lyase
AGC: automatic gain control
AHCYL1: S-adenosylhomocysteine hydrolase-like protein 1
ALDH1B1: aldehyde dehydrogenase X
ALDH18A1: delta-1-pyrroline-5-carboxylate synthase
ALDH2: aldehyde dehydrogenase
ALDH4A1: delta-1-pyrroline-5-carboxylate dehydrogenase
ALDOA/C: fructose-bisphosphate aldolase A/C
APRT: adenine phosphoribosyltransferase
ASL: argininosuccinate lyase
ATP: adenosine triphosphate
CAD: cadherin-1
CHOP: phosphorylcholine
CMP-Neu5Ac: cytidine monophosphate N-acetylneuraminic acid
CS: citrate synthase
CTPs2: CTP synthase 2
DCK: deoxycytidine kinase
DHAP: dihydroxyacetone phosphate
DLAT: dihydrolipoyllysine-residue acetyltransferase component of pyruvate dehydrogenase complex
ENO1/2/3: alpha/gamma/beta-enolase
FAH: fumarylacetoacetase
FH: fumarate hydratase
G3P: 3-phosphoglyceric acid
GAD: glutamate decarboxylase 1
GCLC: glutamate-cysteine ligase catalytic subunit
GFPT1: glutamine-fructose-6-phosphate aminotransferase
GGH: gamma-glutamyl hydrolase
GLS: glutaminase kidney isoform
GLUD1: glutamate dehydrogenase 1
GLUL: glutamine synthetase
GMP: guanosine monophosphoric acid
GMPS: GMP synthase
GNPAT: dihydroxyacetone phosphate acyltransferase
GOT: aspartate aminotransferase
GPC: glycerophosphocholine
HPRT1: hypoxanthine-guanine phosphoribosyltransferase
LDHA: L-lactate dehydrogenase A
NANS: sialic acid synthase
NCE: normalized collisional energy
ME2: NAD-dependent malic enzyme
MTAP: S-methyl-5'-thioadenosine phosphorylase
OPLS-DA: orthogonal partial least square discriminant analysis
PC: phosphatidylcholine
PCA: principal component analysis
PCK2: phosphoenolpyruvate carboxykinase
PDHA1: pyruvate dehydrogenase E1 component subunit alpha
PDHB: pyruvate dehydrogenase E1 component subunit beta
PDP1: pyruvate dehydrogenase-phosphatase 1
PEP: phosphoenolpyruvic acid
PKLR: pyruvate kinase PKLR
PKM: pyruvate kinase PKM
PNP: purine nucleoside phosphorylase
PSAT1: phosphoserine aminotransferase
SAM: S-adenosylmethionine
SDHA: succinate dehydrogenase flavoprotein subunit
SDHB: succinate dehydrogenase iron-sulfur subunit
SDHAF2: succinate dehydrogenase assembly factor 2
TCA cycle: tricarboxylic acid cycle
UDP: uridine 5'-diphosphate
UDP-GlcNAc: uridine diphosphate-N-acetylglucosamine
UDP-Neu5Ac: N-acetylneuraminic acid
UMP: uridine 5'-monophosphate
VIP: variable importance in the projection

## References

[1] Siegel RL, Miller KD, Jemal A (2020). Cancer statistics, 2020. CA Cancer J Clin.

[2] Akiyama T, Sudo C, Ogawara H (1986). The product of the human c-erbB-2 gene: a 185-kilodalton glycoprotein with tyrosine kinase activity. Science.

[3] Vernieri C, Milano M, Brambilla M (2019). Resistance mechanisms to anti-HER2 therapies in HER2-positive breast cancer: Current knowledge, new research directions and therapeutic perspectives. Crit Rev Oncol Hematol.

[4] Chen R, Lai LA, Sullivan Y (2017). Disrupting glutamine metabolic pathways to sensitize gemcitabine-resistant pancreatic cancer. Sci Rep.

[5] Zhou Y, Wang K, Zhou Y (2020). HEATR1 deficiency promotes pancreatic cancer proliferation and gemcitabine resistance by up-regulating Nrf2 signaling. Redox Biol.

[6] Liu W, Yuan J, Liu Z (2018). Label-free quantitative proteomics combined with biological validation reveals activation of Wnt/*β*-catenin pathway contributing to trastuzumab resistance in gastric cancer. Int J Mol Sci.

[7] Sulahian R, Casey F, Shen J (2014). An integrative analysis reveals functional targets of GATA6 transcriptional regulation in gastric cancer. Oncogene.

[8] Song Y, Tian T, Fu X (2015). GATA6 is overexpressed in breast cancer and promotes breast cancer cell epithelial-mesenchymal transition by upregulating slug expression. Exp Mol Pathol.

[9] Lin L, Bass AJ, Lockwood WW (2012). Activation of GATA binding protein 6 (GATA6) sustains oncogenic lineage-survival in esophageal adenocarcinoma. Proc Natl Acad Sci.

[10] Kamnasaran D, Qian B, Hawkins C (2007). GATA6 is an astrocytoma tumor suppressor gene identified by gene trapping of mouse glioma model. Proc Natl Acad Sci.

[11] Chang J, Wang Y, Zhang F (2019). Proteomic study of transcription factors in trastuzumab-resistant gastric cancer based on liquid chromatography-mass spectrometry technique. Chinese J Anal Chem.

[12] Liu W, Yuan J, Chang J (2020). Label-free quantitative proteomics for investigation of signaling pathways of GATA6 regulating trastuzumab resistance in gastric cancer cells. Chinese J Anal Chem.

[13] Shi Y, Wang Y, Huang W (2019). Integration of metabolomics and transcriptomics to reveal metabolic characteristics and key targets associated with cisplatin resistance in non-small cell lung cancer. J Proteome Res.

[14] Liu W, Wang Q, Chang J (2019). Global metabolomic profiling of trastuzumab resistant gastric cancer cells reveals major metabolic pathways and metabolic signatures based on UHPLC-Q Exactive-MS/MS. RSC Adv.

[15] Smith CA, Want EJ, O'Maille G (2006). XCMS: processing mass spectrometry data for metabolite profiling using nonlinear peak alignment, matching, and identification. Anal Chem.

[16] Bijlsma S, Bobeldijk L, Verheij ER (2006). Large-scale human metabolomics studies: a strategy for data (pre-) processing and validation. Anal Chem.

[17] Chong J, Soufan O, Li C (2018). MetaboAnalyst 4.0: towards more transparent and integrative metabolomics analysis. Nucl Acids Res.

[18] Zhou G, Xia J (2019). Using OmicsNet for network integration and 3D visualization. Curr Protoc Bioinformatics.

[19] Szklarczyk D, Gable AL, Lyon D (2019). STRING v11: protein-protein association networks with increased coverage, supporting functional discovery in genome-wide experimental datasets. Nucl Acids Res.

[20] Anderson NM, Mucka P, Kern JG (2018). The emerging role and targetability of the TCA cycle in cancer metabolism. Protein Cell.

[21] Ren J, Seth P, Clish CB (2014). Knockdown of malic enzyme 2 suppresses lung tumor growth, induces differentiation and impacts PI_3_K/AKT signaling. Sci Rep.

[22] Sarfraz I, Rasul A, Hussain G (2018). Malic enzyme 2 as a potential therapeutic drug target for cancer. IUBMB Life.

[23] Pavlova NN, Thompson CB (2016). The emerging hallmarks of cancer metabolism. Cell Metab.

[24] Zhao H, Duan Q, Zhang Z (2017). Up-regulation of glycolysis promotes the stemness and EMT phenotypes in gemcitabine-resistant pancreatic cancer cells. J Cell Mol Med.

[25] Zhao Y, Zhou M, Liu H (2009). Upregulation of lactate dehydrogenase a by ErbB2 through heat shock factor 1 promotes breast cancer cell glycolysis and growth. Oncogene.

[26] Fantin VR, St-Pierre J, Leder P (2006). Attenuation of LDH-A expression uncovers a link between glycolysis, mitochondrial physiology, and tumor maintenance. Cancer Cell.

[27] Wang Y, Fan S, Lu J (2017). GLUL promotes cell proliferation in breast cancer. J Cell Biochem.

[28] Cheyne RW, Trembleau L, McLaughlin A (2011). Changes in 2-fluoro-2-deoxy-D-glucose incorporation, hexokinase activity and lactate production by breast cancer cells responding to treatment with the anti-HER-2 antibody trastuzumab. Nucl Med Biol.

[29] Wei Tan H, Ning Leung CO, Shuen Chan KK (2019). Deregulated GATA6 modulates stem cell-like properties and metabolic phenotype in hepatocellular carcinoma. Int J Cancer.

[30] Miao P, Sheng S, Sun X (2013). Lactate dehydrogenase A in cancer: a promising target for diagnosis and therapy. IUBMB Life.

[31] Zhou M, Zhao Y, Ding Y (2010). Warburg effect in chemosensitivity: targeting lactate dehydrogenase-A re-sensitizes taxol-resistant cancer cells to taxol. Mol Cancer.

[32] Lukey MJ, Katt WP, Cerione RA (2017). Targeting amino acid metabolism for cancer therapy. Drug Discov Today.

[33] Altman BJ, Stine ZE, Dang CV (2016). From Krebs to clinic: glutamine metabolism to cancer therapy. Nat Rev Cancer.

[34] Bott AJ, Maimouni S, Zong W (2019). The pleiotropic effects of glutamine metabolism in cancer. Cancers.

[35] Thornburg JM, Nelson KK, Clem BF (2008). Targeting aspartate aminotransferase in breast cancer. Breast Cancer Res.

[36] Hong C, Zheng J, Li X (2017). Inhibition of GOT1 sensitizes colorectal cancer cells to 5-fluorouracil. Cancer Chemother Pharmacol.

[37] Fu A, Yu Z, Song Y (2015). Silencing of glutaminase 1 re-sensitizes taxol-resistant breast cancer cells to taxol. Mol Med Rep.

[38] Santana-Codina N, Roeth AA, Zhang Y (2018). Oncogenic KRAS supports pancreatic cancer through regulation of nucleotide synthesis. Nat Commun.

[39] Cory JG, Cory AH (2006). Critical roles of glutamine as nitrogen donors in purine and pyrimidine nucleotide synthesis: Asparaginase treatment in childhood acute lymphoblastic leukemia. *In vivo*.

[40] Peng Y, Fu S, Hu W (2020). Glutamine synthetase facilitates cancer cells to recover from irradiation-induced G2/M arrest. Cancer Biol Ther.

