# Supplementary material for: Metabolic pathways underlying GATA6 regulating Trastuzumab resistance in Gastric Cancer cells based on untargeted metabolomics
Source: Int J Med Sci. 2020 Oct 23;17(18):3146–64. doi: 10.7150/ijms.50563 (PMC7646115; doi:10.7150/ijms.50563)
Supplement: Supplementary file 1 — Supplementary figures and tables. [file ijmsv17p3146s1.pdf]

Supplementary materials

Metabolic Pathways Underlying GATA6 Regulating Trastuzumab Resistance in Gastric Cancer Cells Based on Untargeted Metabolomics

Jinxia Chang [1*], Qiang Wang [3*], Anup Bhetuwal [4], and Wenhu Liu [1,2] ✉

1. School of Basic Medical Sciences, North Sichuan Medical College, Nanchong, Sichuan 637100, China
2. School of Pharmacy, North Sichuan Medical College, Nanchong, Sichuan 637100, China
3. Department of Laboratory Medicine, Affiliated Hospital of North Sichuan Medical College; Faculty of Laboratory Medicine, Center for Translational Medicine, North Sichuan Medical College, Nanchong, Sichuan 637000, China
4. Sichuan Key Laboratory of Medical Imaging and Department of Radiology, Affiliated Hospital of North Sichuan Medical College, Nanchong, Sichuan 637000, China

[*]These authors contributed equally to this work.

✉ Corresponding author: Wenhu Liu, Ph.D., School of Pharmacy, North Sichuan Medical College, 55 Dongshun Rd. Nanchong, Sichuan 637100, China, E-mail: wenhuliu@cqu.edu.cn

Fig. S1 PCA-X one-dimensional line plots from QC samples were calculated to assess the experiment reproducibility of trastuzumab resistant groups and GATA6 knock out groups in ESI+ and ESI– respectively.

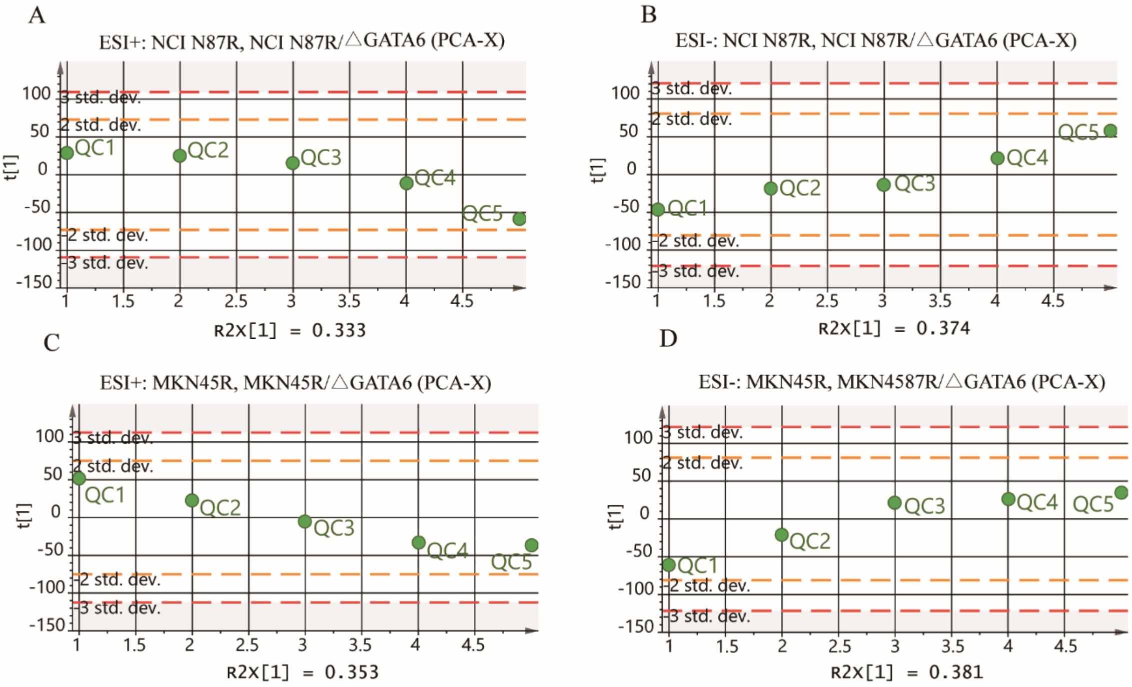

Fig. S2 Spearman's correlation coefficients from QCs samples were calculated to assess the experiment reproducibility of NCI N87R and NCI N87R/ΔGATA6 cells in ESI+ (A) and ESI– (B), and MKN45R and MKN45R/ΔGATA6 cells in ESI+ (C) and ESI– (D) respectively. The lower-left half shows pairwise scatter plots of QCs samples. The upper-right half shows pairwise Spearman's correlation coefficients from the same comparison.

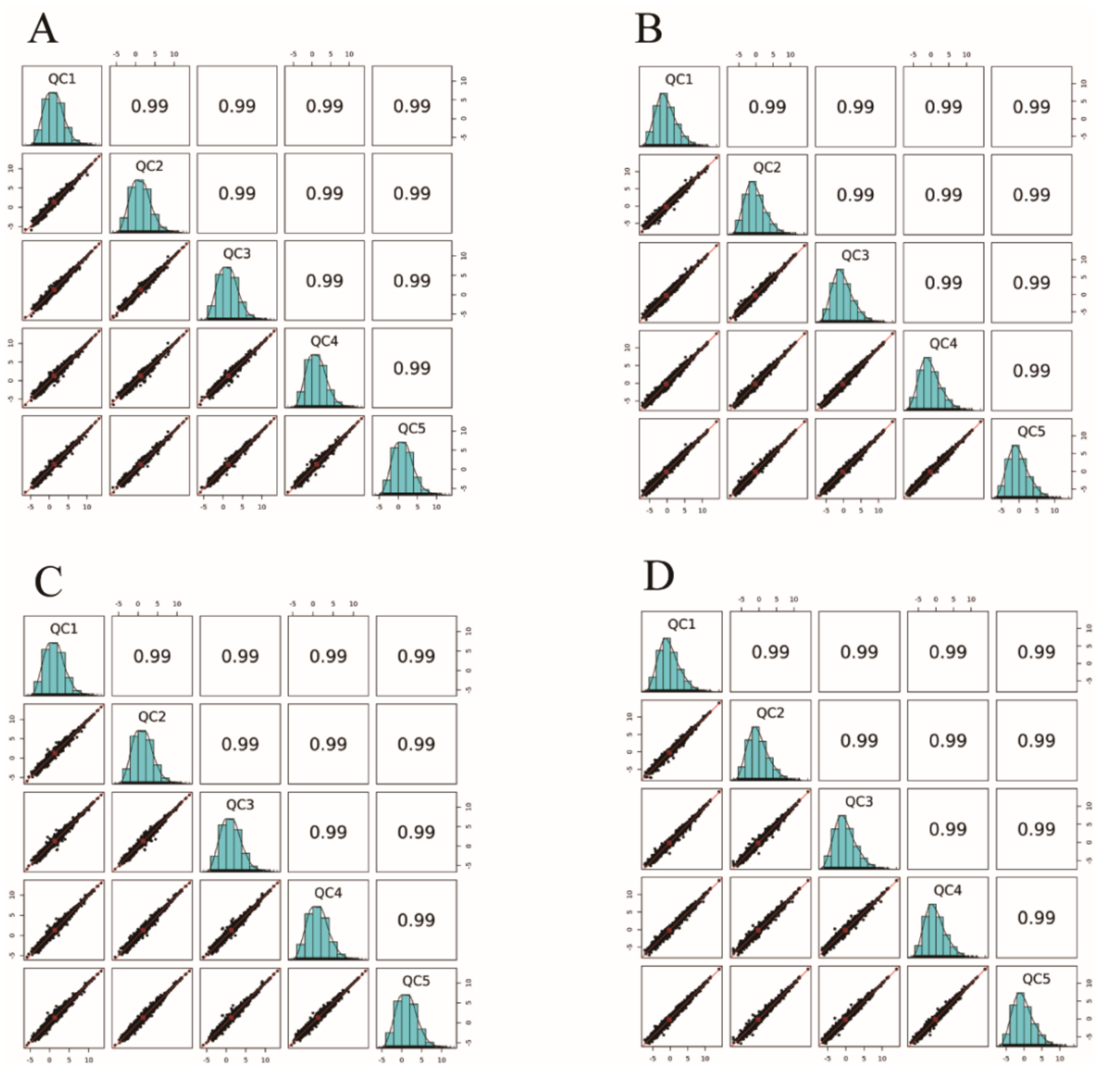

Fig. S3 Hierarchical clustering analysis of NCI N87R and NCI N87R/ΔGATA6 cells (A, B), MKN45R and MKN45R/ΔGATA6 cells (C, D) in ESI+ and ESI– respectively. The increased and decreased features are represented by range of red and blue intensities, respectively.

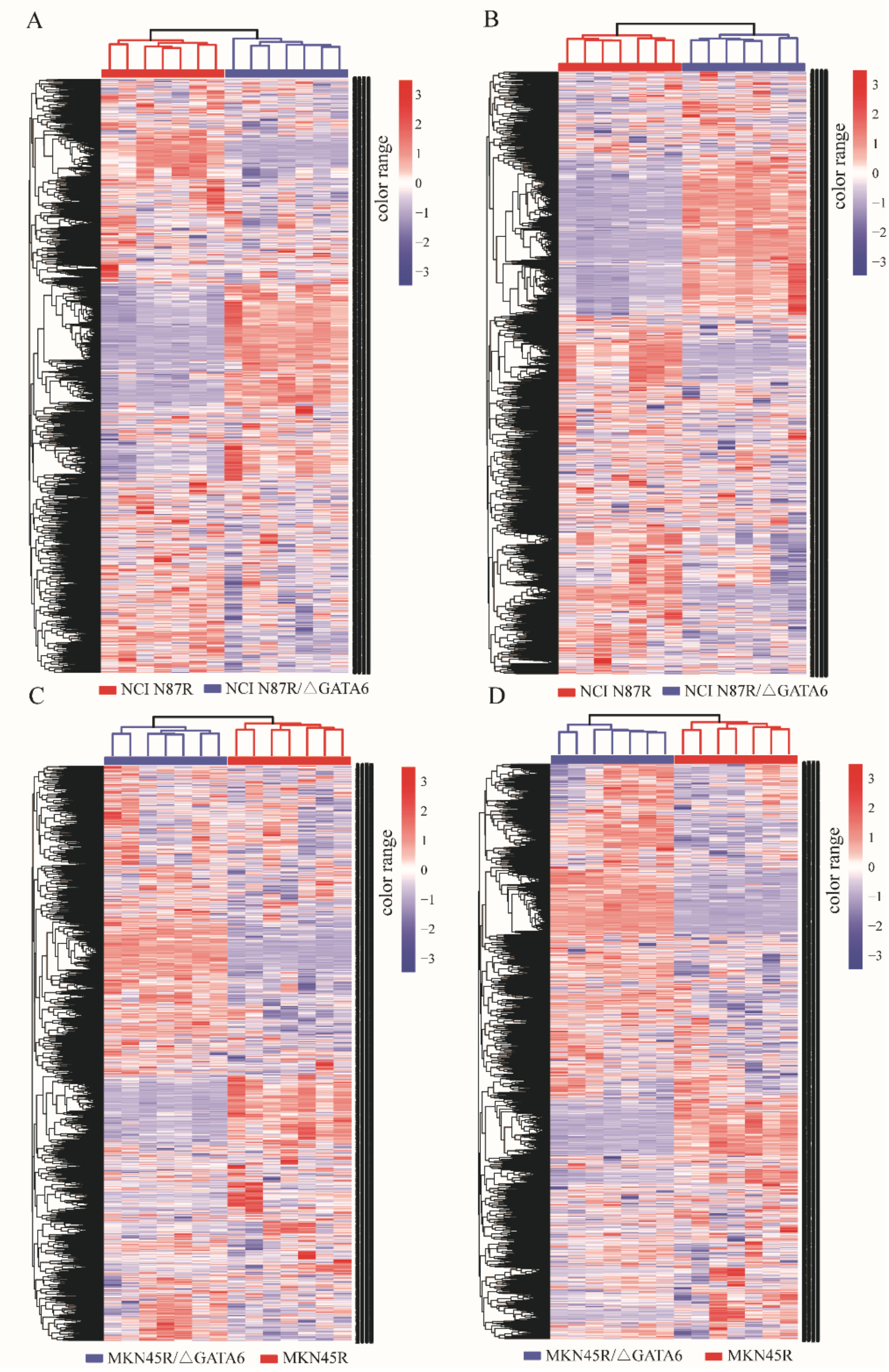

Fig. S4 (A-D) Differential metabolites among NCI N87R, NCI N87R/ΔGATA6, MKN45R and MKN45R/ΔGATA6 cells are illustrated by volcano plots in ESI+ and ESI-, respectively. The mean ratios of seven biological repeats were plotted in log2 scale (x-axis) against the corresponding-log10 *p* value (y-axis). The vertical dotted lines mark 1.2 and 0.83 fold change while horizontal dotted lines represent cutoff p=0.05. Metabolites with fold changes greater than 1.2 or less than 0.83 and *p*<0.05 are regarded as increased or decreased and marked in red and blue, respectively. The gray dots are considered as having no significant change.

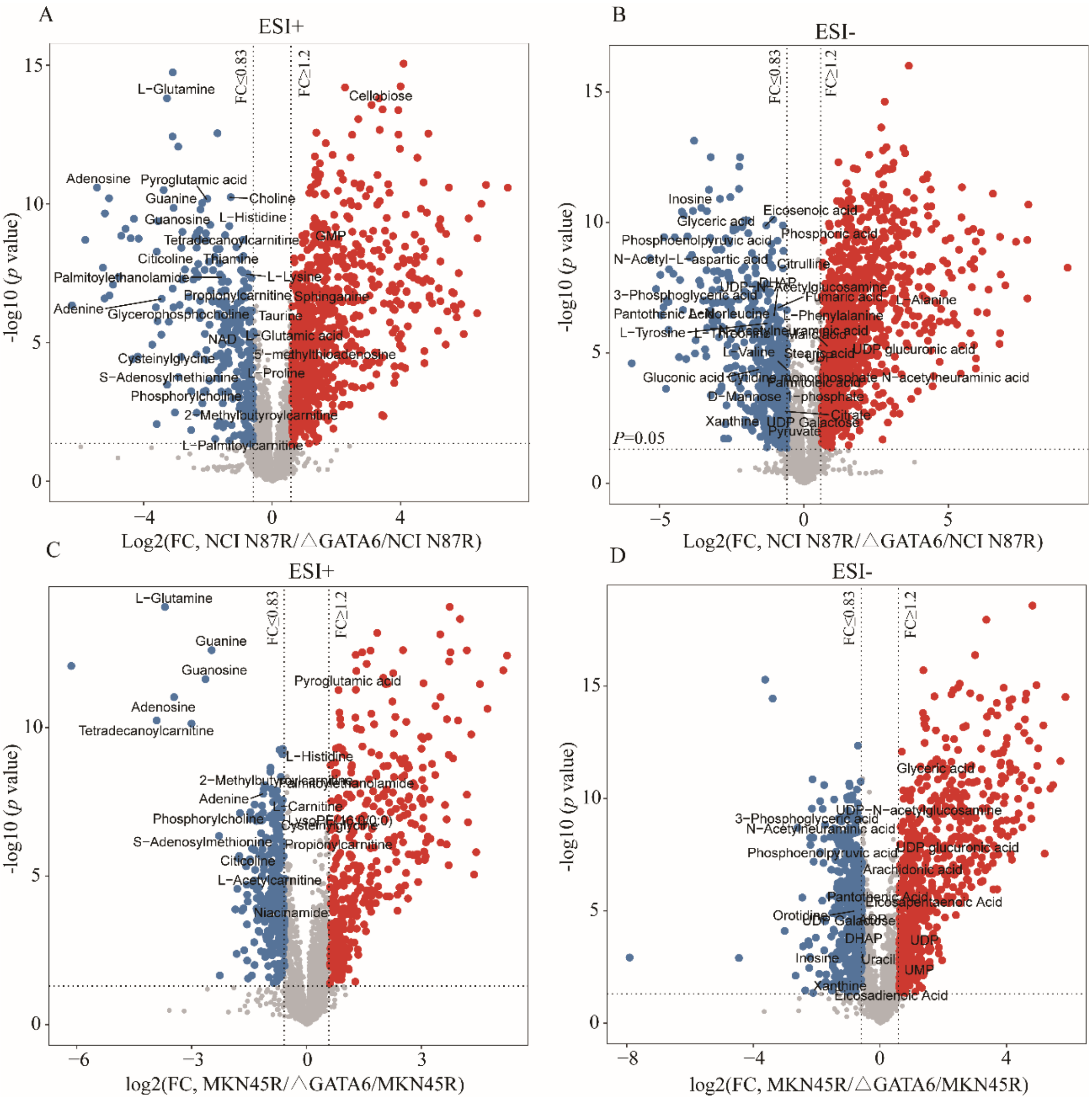

Fig. S5 Violin plots of the differential metabolites were showed in NCI N87R/ΔGATA6 *vs*. NCI N87R cells.

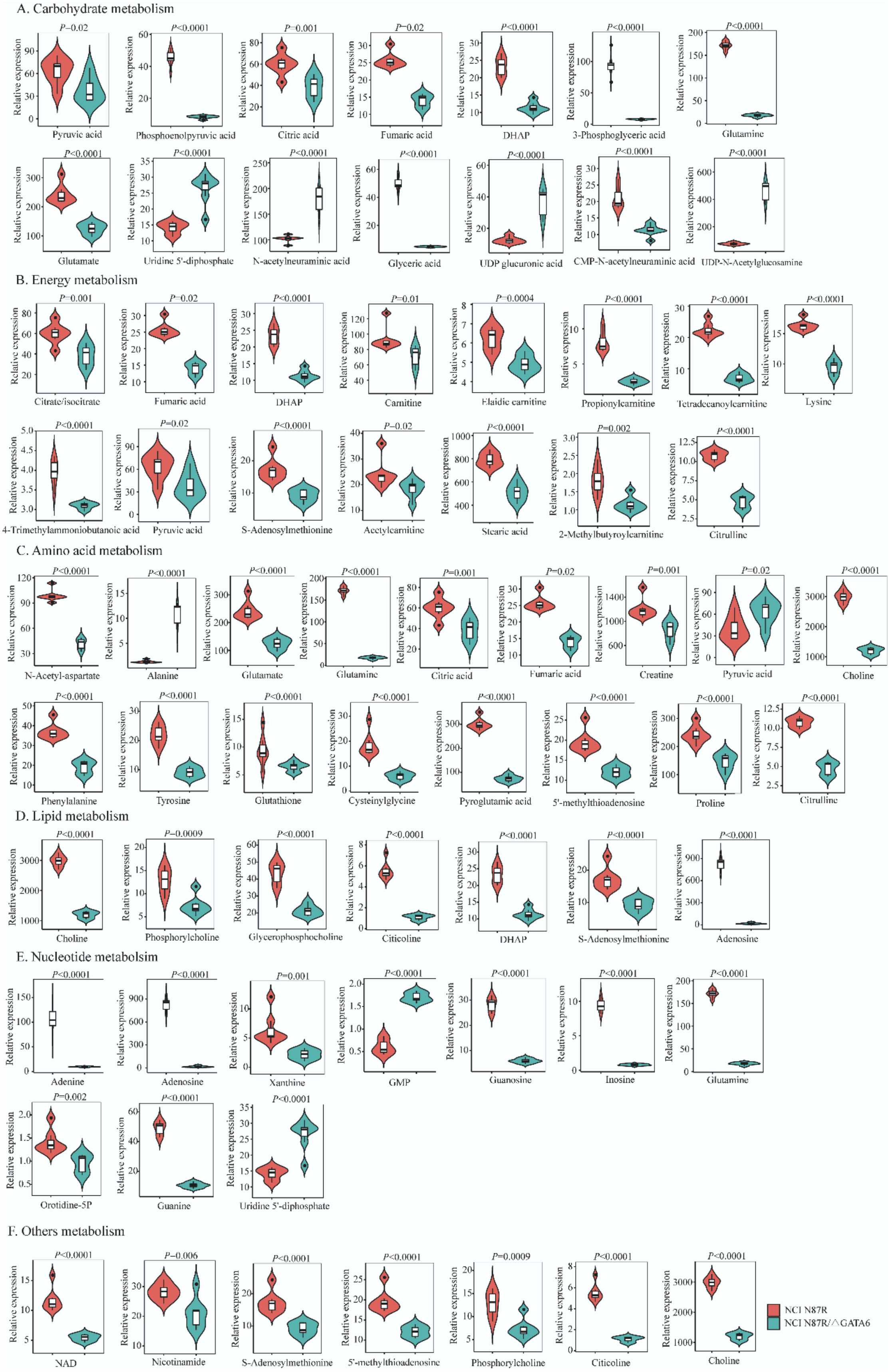

Fig. S6 Violin plots of the differential metabolites were showed in MKN45R/ΔGATA6 *vs.* MKN45R cells.

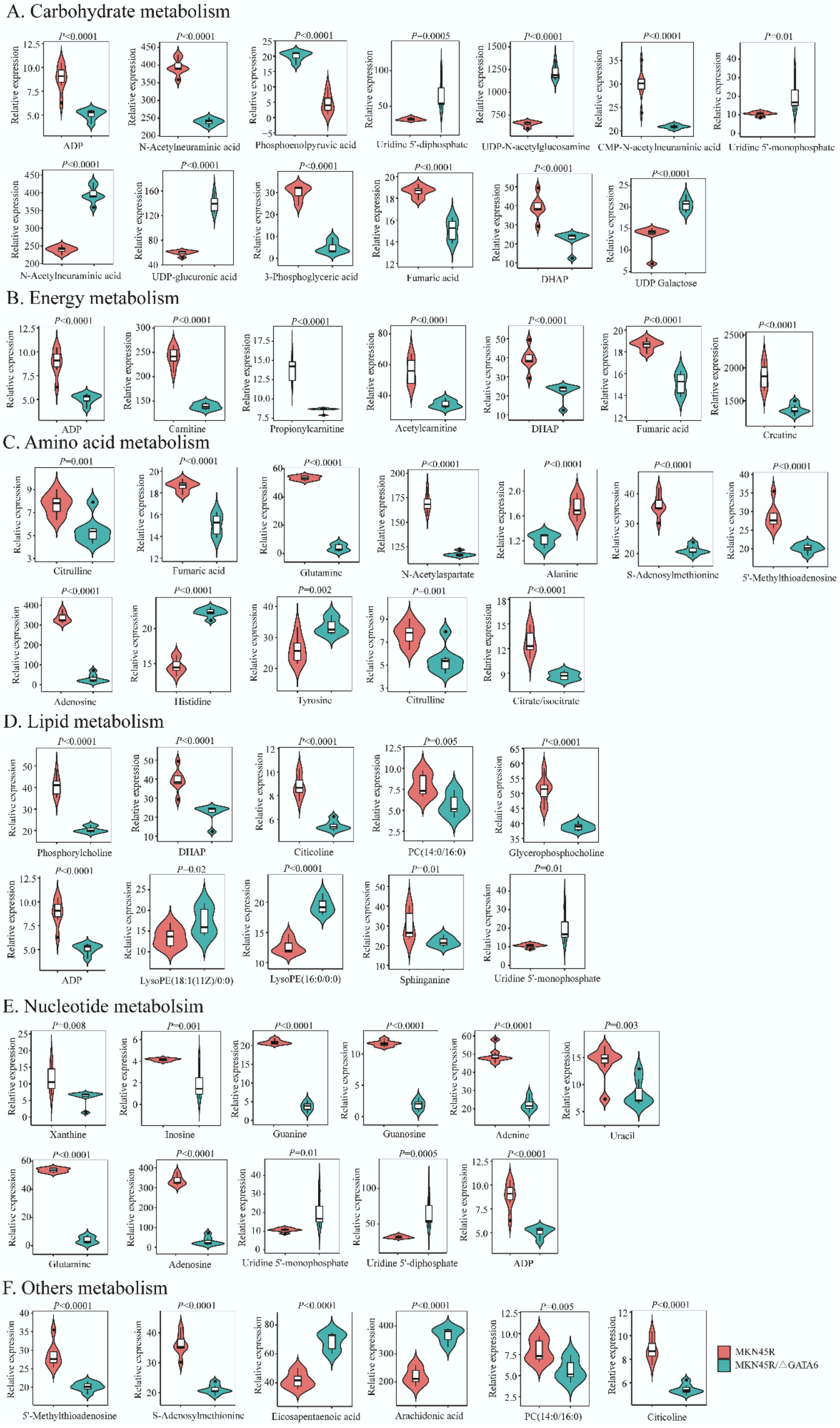

Table. S1 Response stability of internal standard from QC samples

Internal standard	ESI+			ESI–		
2-chloro-L-phenylalanine	m/z	RT(s)	RSD	m/z	RT(s)	RSD
	200.0663	175.5319	3.24%	198.142	165.825	4.79%

Table S2. Differential metabolites overlapped in NCI N87R, NCI N87R/ΔGATA6 and MKN45R, MKN45R/ΔGATA6 cells

No	Metabolites	HMDB	KEGG	Tread (NCI N87R/ΔGATA6/NCI N87R)	Tread (MKN45R/ΔGATA6/MKN45R)
1	5'-Methylthioadenosine	HMDB0001173	C00170	Down	Down
2	Gluconic acid	HMDB0000625	C00257	Down	Up
3	Xanthine	HMDB0000292	C00385	Down	Down
4	L-Tyrosine	HMDB0000158	C00082	Down	Up
5	Sphinganine	HMDB0000269	C00836	Up	Down
6	Creatine	HMDB0000064	C00300	Down	Down
7	L-Acetylcarnitine	HMDB0000201	C02571	Down	Down
8	Glycerophosphocholine	HMDB0000086	C00670	Down	Down
9	Fumaric acid	HMDB0000134	C00122	Down	Down
10	Guanosine	HMDB0000133	C00387	Down	Down
11	Pyroglutamic acid	HMDB0000267	C01879	Down	Up
12	Niacinamide	HMDB0001406	C00153	Down	Down
13	Cysteinylglycine	HMDB0000078	C01419	Down	Down
14	S-Adenosylmethionine	HMDB0001185	C00019	Down	Down
15	Glyceric acid	HMDB0000139	C00258	Down	Up
16	Palmitoylethanolamide	HMDB0002100	C16512	Down	Up
17	Tetradecanoylcarnitine	HMDB0005066	—	Down	Down
18	Phosphorylcholine	HMDB0001565	C00588	Down	Down
19	Citrulline	HMDB0000904	C00327	Down	Down
20	L-Histidine	HMDB0000177	C00135	Down	Up
21	N-Acetyl-L-aspartic acid	HMDB0000812	C01042	Down	Down
22	Adenosine	HMDB0000050	C00212	Down	Down
23	Adenine	HMDB0000034	C00147	Down	Down
24	UDP Galactose	HMDB0000302	C00052	Up	Up
25	Inosine	HMDB0000195	C00294	Down	Down
26	Phosphoenolpyruvic acid	HMDB0000263	C00074	Down	Down
27	Pantothenic acid	HMDB0000210	C00864	Down	Down
28	2-Methylbutyroylcarnitine	HMDB0000378	—	Down	Down
29	L-Alanine	HMDB0000161	C00041	Up	Up
30	L-Glutamine	HMDB0000641	C00064	Down	Down
31	Malic acid	HMDB0000744	C03668	Down	Down
32	L-Threonine	HMDB0000167	C00188	Down	Up
33	Orotidine	HMDB0000788	C01103	Down	Down
34	3-Phosphoglyceric acid	HMDB0000807	C00597	Down	Down
35	CMP-Neu5Ac	HMDB0001176	C00128	Down	Down
36	L-Carnitine	HMDB0000062	C00318	Down	Down
37	Guanine	HMDB0000132	C00242	Down	Down
38	UDP glucuronic acid	HMDB0000935	C00167	Up	Up
39	N-Acetylneuraminic acid	HMDB0000230	C19910	Up	Down

40	Propionylcarnitine	HMDB0000824	C03017	Down	Down
41	D-Mannose 1-phosphate	HMDB0006330	C00636	Down	Up
42	Uridine 5'-diphosphate	HMDB0000295	C00015	Up	Up
43	Dihydroxyacetone phosphate	HMDB0001473	C00111	Down	Down
44	Eicosenoic acid	HMDB0002231	C16526	Down	Up
45	Citicoline	HMDB0001413	C00307	Down	Down
46	UDP-N-Acetylglucosamine	HMDB0000290	C00043	Up	Down
47	Citrate/isocitrate	HMDB0000094	C00158	Down	Down

Table S3. Differential metabolites in NCI N87R/ΔGATA6 *vs*. NCI N87R cells

No	Metabolites	HMDB	KEGG	Tread (NCI N87R/ΔGATA6/NCI N87R)
1	Citrate/isocitrate	HMDB0000094	C00158	Down
2	L-Glutamic acid	HMDB0000148	C00025	Down
3	Cholesterol sulfate	HMDB0000653	C18043	Up
4	NAD	HMDB0000902	C00003	Down
5	4-Trimethylammoniobutanoic acid	HMDB0001161	C01181	Down
6	Palmitoleic acid	HMDB0003229	C08362	Up
7	L-Valine	HMDB0000883	C00183	Down
8	Thiamine	HMDB0000235	C00378	Down
9	L-Proline	HMDB0000162	C00148	Down
10	Phosphoric acid	HMDB0002142	C00009	Down
11	L-Norleucine	HMDB0001645	C01933	Down
12	Taurine	HMDB0000251	C00245	Down
13	GMP	HMDB0001397	C00144	Up
14	Pyruvic acid	HMDB0000243	C00022	Down
15	Glutathione	HMDB0000125	C02471	Down
16	L-Palmitoylcarnitine	HMDB0000222	C02990	Down
17	Cellobiose	HMDB0000055	C06422	Up
18	Stearic acid	HMDB0000827	C01530	Down
19	Choline	HMDB0000097	C00114	Down
20	L-Phenylalanine	HMDB0000159	C00079	Down
21	Elaidic carnitine	HMDB0006464	—	Down
22	L-Lysine	HMDB0000182	C00047	Down

Table S4. Differential metabolites in MKN45R/ΔGATA6 *vs*. MKN45R cells

No	Metabolites	HMDB	KEGG	Tread (MKN45R/ΔGATA6/MKN45R)
1	Eicosapentaenoic acid	HMDB0001999	C06428	Up
2	Arachidonic acid	HMDB0001043	C00219	Up
3	LysoPE (18:1(11Z)/0:0)	HMDB0011505	—	Up
4	PC (14:0/16:0)	HMDB0007869	C00157	Down
5	Lauroyl diethanolamide	HMDB0032358	—	Down
6	Uracil	HMDB0000300	C00106	Down
7	Niacinamide	HMDB0001406	C00153	Down
8	Eicosadienoic acid	HMDB0005060	C16525	Up
9	Glyceraldehyde	HMDB0001051	C02154	Down
10	Uridine 5'-monophosphate	HMDB0000288	C00105	Up
11	LysoPE (16:0/0:0)	HMDB0011503	—	Up
12	ADP	HMDB0001341	C00008	Down
